# Lignan enriched fraction (LRF) of *Phyllanthus amarus* promotes apoptotic cell death in human cervical cancer cells *in vitro*

**DOI:** 10.1038/s41598-019-51480-7

**Published:** 2019-10-18

**Authors:** Subhabrata Paul, Debashis Patra, Rita Kundu

**Affiliations:** 1 Bioprospecting Laboratory, School of Biotechnology, Department of Life Sciences, Presidency University, Kolkata, India; 2 Chemistry Laboratory, Department of Science & Humanities, Acharya Jagadish Chandra Bose Polytechnic, Berachampa, West Bengal India; 3 Cell Biology Laboratory, Department of Botany, Centre of Advanced Studies, University of Calcutta, Kolkata, India

**Keywords:** Apoptosis, Stress signalling

## Abstract

*Phyllanthus amarus* is widely grown in this sub-continent and used traditionally to treat many common ailments. In the present study, lignan rich fraction of *P*. *amarus* extract was used on cervical cancer cell lines (HeLa, SiHa and C33A) to study it’s mechanism of cell death induction. As the cells were treated with IC50 doses of LRF, characteristic apoptotic features were observed. Increased sub G0 population were observed both in Hela and C33 cells, while G1/S arrest was observed in SiHa cells than their untreated counterparts. Increased production of ROS and change in MMP were also detected in the treated cells. Presence of γH2AX, was observed by immunofluorescence. Reduced expression of HPV (16/18) as well as ET-1, an autocrine growth substance, were observed in the treated cells. Immunoblotting as well as ICFC studies showed enhanced expressions of BAX, Caspase 3 and PARP (cleaved) in the treated cells. A major lignan, phyllanthin was isolated from the chloroform fraction and showed strong irreversible affinities for viral E6 and MDM2 in *in silico* analysis. The study conclusively indicates that LRF has the potential to induce apoptotic cell death in cervical cancer cells by activation of p53 and p21 against DNA damage.

## Introduction

With approximately 569,847 new cases and 311,365 deaths, cervical cancer ranks fourth in cancer related death worldwide^1^. In countries having lower HDI (Human Development Index), it is second in position among women, both in incidence as well as in death rate. All over the world, cervical cancer possess a great threat to women, it is the leading cause of death in 42 countries while, there are nearly 30 countries where this is the most common cancer among the women.The majority areas afflicted with this cancer are the Sub-Saharan Africa, and South Eastern Asia. Highest rate of incidence and mortality are recorded from Eastern and Western Africa. Comparatively lesser incidences and mortality are reported from Australia, North America and Western Asia^1^. Majority of the cervical cancer cases (more than 70%) showed presence of human papillomavirus such as HPV 16 and HPV 18 which are considered as high risk ones. In cervical cancer, p53 is usually wild type^2^. The viral E6 protein principally targets the cellular tumor suppressor p53 protein and inactivates it to establish itself. In HPV-bearing cancer cells, E6 oncoprotein ubiquitinates the functional p53 protein, affects its stability and degrades it rapidly via cellular ubiquitin ligase. This degradation is similar to an inactivating mutation which in turn increases cell proliferation by abrogating normal functions of p53, like G1 arrest, DNA repair and apoptosis^2^. ET-1 activates the autocrine and paracrine signalling in HPV infected cells and modulates several cellular functions like cell proliferation, EMT, cell migration, apoptosis and chemo-resistance^3^.

Chemotherapy and radiation therapy are the standard treatment procedures used against cancer till now. Avastin, Bleomycin, Topotecan and Cisplatin-Gemcitabine combination are the approved chemotherapeutic drugs generally used to treat cervical cancer. Avastin inhibits angiogenesis by binding directly with vascular endothelial growth factor (VEGF). Bleomycin inhibits DNA synthesis while topotecan induces single stranded DNA damage and apoptosis. Cisplatin induces DNA damage and interferes with DNA replication. Gemcitabine inhibits DNA synthesis and induces cytotoxicity. Though the combinatorial therapy proved to be efficient, acquired chemoresistance of the cancer cells or treating immune-compromised patients yields poor response. Moreover, chemotherapy is associated with several side effects like, cognitive impairment, sensory impairment, organ damage (lung, liver, neural), gastrointestinal disorders, infertility, hair loss, fatigue, etc. On the other hand, radiation has the problem of non-selective cytotoxicity leading to immunotoxicity^4^. Continuous emergence of drug resistance against drugs in practice is another hurdle in the way^5^.That is why finding new anticancer compounds should go hand in hand with basic cancer biology research; the most common approach is screening new synthetic/semi-synthetic compounds or modification of existing compounds using combinatorial chemistry with *in silico* analysis. Another established approach is random screening of natural compounds. In this respect plants are the largest repertoire of various kinds of natural compounds. Traditionally plants are usually utilized for Indian and Chinese herbal drug preparations. According to World Health Organization (WHO), around 33% of anticancer drugs are plant-derived^6^. India, with a rich biodiversity, has ample native plant resources, around 17000 species, out of which around 7000 species are considered to be medicinally important^7^. *Phyllanthus amarus* Schum & Thorn. (popularly known as Sleeping plant) is a small herb, belonging to Euphorbiaceae family. This plant is highly valued in Indian Ayurveda system for its medicinal properties. It is commonly used to treat several common gastrointestinal disorders, like jaundice, diarrhoea, dysentery and wound, ulcers and urogenital diseases^8,9^.

Several phytochemicals, such as, tannins, ellagitannins, triterpenes, flavonoids and alkaloids are present in this plant. The principal secondary metabolites of this plant are bioactive lignans, especially phyllanthin and hypophyllanthin. Lignan rich foods are considered to be advantageous for human health, breast cancer patients with higher lignan intake through food showed better survival chance and reduction in tumor growth^10^.

In the present study, methanolic extract was fractionated and chemically characterized for the presence of the major phytochemicals by chromatographic methods. This lignan rich fraction was used on HeLa (HPV 18 +ve), SiHa (HPV 16 +ve) and C33 A (HPV −ve), three different cervical cancer cells, and efficacy of the LRF on the cell lines was studied. Along with that, cell death induction pathways in the three different cell lines were also evaluated. Apoptosis is induced through extrinsic and /intrinsic pathway. In both cases mitochondria play an important role, triggering enhancement of the pro-apoptotic proteins and reduction of anti-apoptotic ones. So, expression of genes and proteins relevant to apoptosis were studied. As the plant extract is rich in lignans, generation of ROS and subsequent loss in MMP were also studied.

## Results

### Elucidation of chemicals constituents

**Figure 1:**
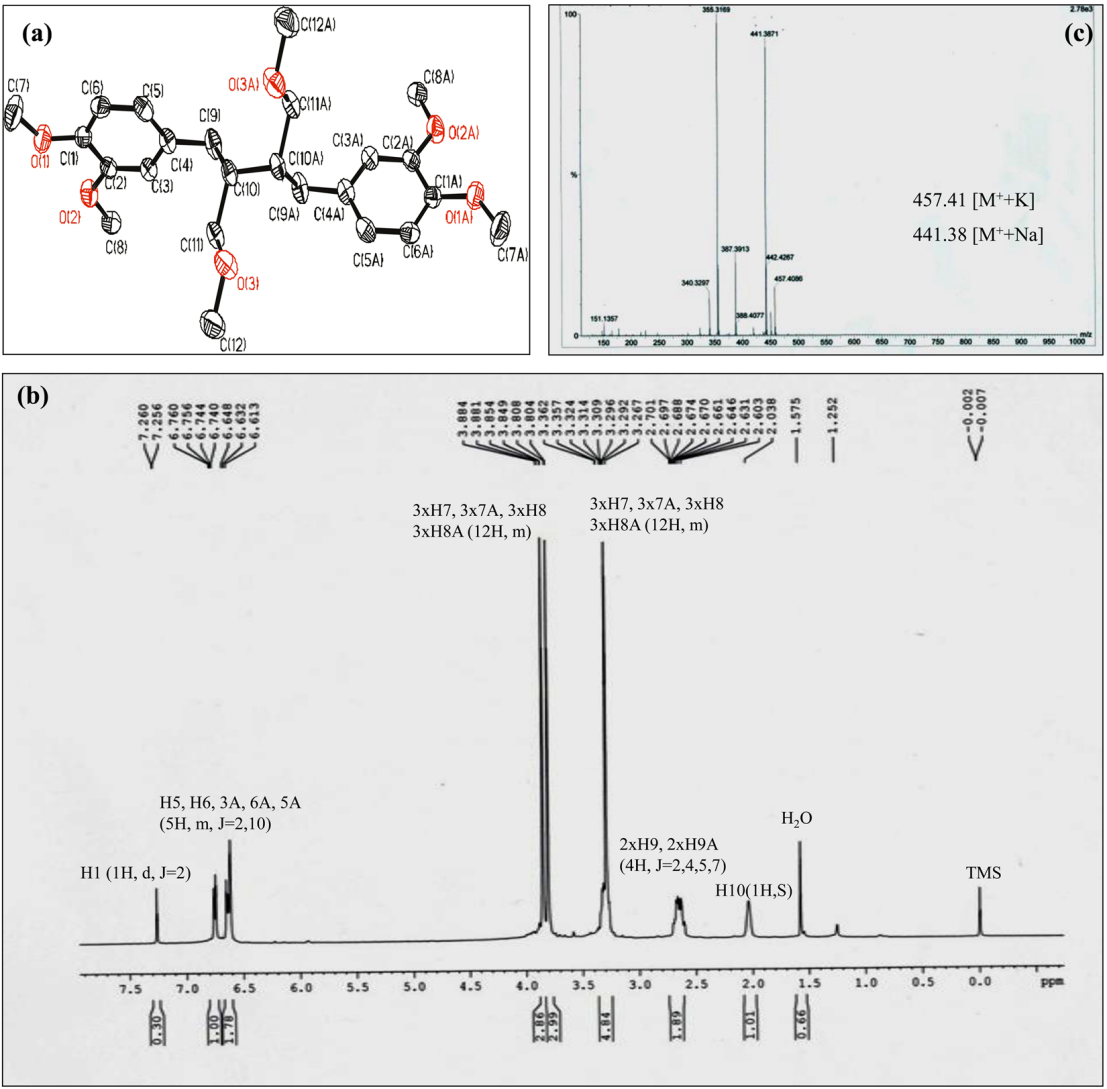
Characterization of Phyllanthin crystal (**a**) XRD analysis showing the crystal structure (**b**) 1H-NMR (**c**) ESI-MS showing the molecular mass.

Several compounds were identified by LC-MS (Table S1 and Fig. S2) using popular databases (ReSpect, MassBank, MZ cloud etc.) comparing monoisotopic mass and fragmentation patterns. Niranthin (a lignan), corilagin (an ellagitannin), rutin and quercetin (flavonoid) are worth mentioning among the identified compounds, as their anti-cancer activities are already reported^11–14^. Several phenolic compounds, terpenoids, specially, squalene (a precursor of steroid biosynthesis) were detected in the LRF by GC-MS analysis (Table S1). Several sub-fractions had also been isolated from LRF by silica gel chromatography. GC-MS analysis of the sub fractions detected methyl esters of several usual phytochemicals such as Palmitic acid, Stearic acid and α-Linolenic acid (as identified with NIST library) after derivatization with TMS. Single crystals isolated by column chromatography were identified as phyllanthin by XRD analysis (CCDC submission ID: 1820905) (Table S2). 1H-NMR and ESI-MS analysis also confirmed that (Fig. 1).

### Evaluation of ROS mediated DNA damage by LRF

#### Flow cytometric determination of ROS induction

**Figure 2:**
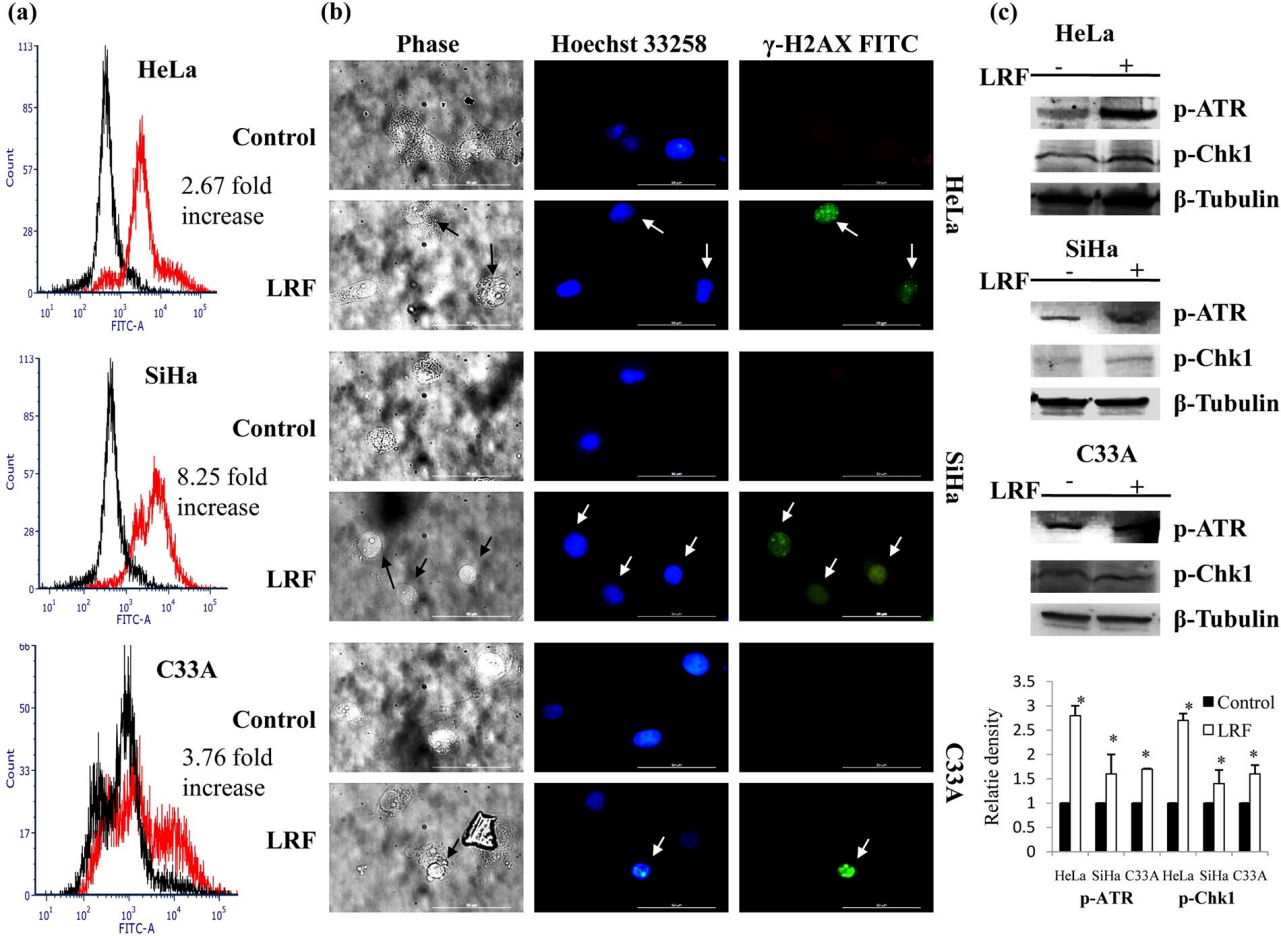
DNA damage as induced by ROS due to LRF treatment. (**a**) Overlapping histograms showing increase of ROS in LRF treated cells. Black and red lines indicate control and treated sets respectively, (**b**) Representative photomicrographs showing incorporations of γ-H2AX in the damaged nuclei. Arrows indicate damaged nuclei (**c**) Representative immunoblots showing increase in expressions of DNA damage responsive proteins. Fold changes (densitometry) were expressed as histograms. Columns represent average of relative densities while bars represent standard deviations. *Denotes significant difference between control and treated sets (P < 0.05).

All the treated cells showed enhanced ROS accumulation after three hours. Maximum ROS was generated in the treated SiHa cells, whereas, Hela cell line showed minimum accumulation of ROS,with control sets showing 7.79% and LRF treated sets with 20.82% ROS positive cell population. A 2.67 fold increase in ROS positive cells were detected in HeLa cells. While in untreated SiHa cells, 6.96% cells were detected with ROS, but, quite a higher percentage of (57.45%) ROS positive cells were detected in the treated sets. Thus, there is an increase of 8.25 fold in SiHa cells. A 3.76 fold increase in cell population was found in C33A cells with 8.19% cells in control sets and 30.83 cells in treated sets (Fig. 2a).

#### DNA damage detection by γ-H2AX incorporation

Reactive oxygen Species (ROS) can induce DNA damage, resulting either single /double stranded nicks. As the nicks are generated, the H2AX (a variant of H2A) becomes phosphorylated and spreads over the nicks, which are known as foci. Immunofluorescence micrographs clearly indicated the incorporation of phosphorylated H2AX(γ-H2AX) in nuclei of LRF treated cell. Incorporation of γ-H2AX is a clear marker of single or double stranded DNA damage (Fig. 2b) and apoptosis.

#### Study of DNA damage response by immunoblotting

Expressions of proteins as induced by DNA damage, were also studied by western blotting to validate this. In all the treated cells phospho-ATM and phospho-Chk2 could not be detected, but presence of phospho-ATR and phospho-Chk1were detected indicating activation of checkpoint-kinase 1 and ATR. In the treated HeLa cells, a significant increase in the expression of p-ATR and p-Chk1 (1.8 fold and 1.7 fold respectively) were observed. But, in treated SiHa cells, the increase was comparatively less (0.6 fold and 0.4 fold) for p-ATR and p-Chk1 respectively. In case of treated C33A cells, however, p-ATR and p-Chk1 expressions were increased by 0.7 fold and 0.6 fold respectively (Fig. 2c).This clearly indicate that, LRF induced ROS can cause DNA damage in these cancer cells.

### Determination of Apoptosis inducing potential of LRF

#### Apoptosis induction

**Figure 3:**
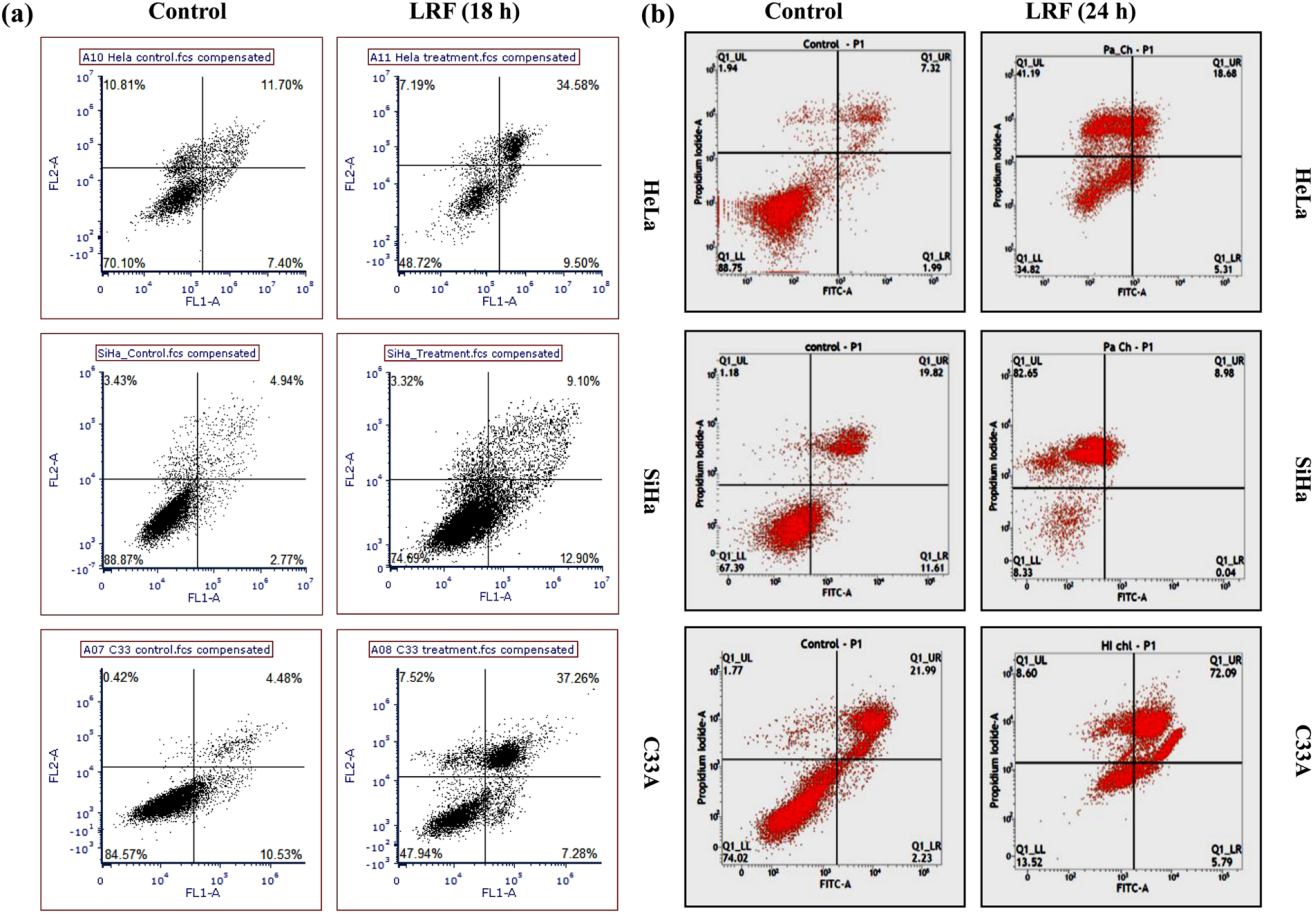
Flow cytometric analysis showing apoptosis inducing activity of LRF in cervical cancer cells at 18 h (**a**) and 24 h (**b**) in Hela, SiHa and C33A cells respectively; in section (**a**) X axis (FL1-A) corresponds to FITC and Y axis (FL2-A) corresponds to PI.

At 18 h treatment time, most of the cells showed signs of later stage of apoptosis. These observations clearly indicate towards induction of apoptosis in the treated cells (Fig. 3a). In HeLa cells, 45.54% cells were found to be apoptotic, with (7.19%) dead cells. In SiHa cells, 22% apoptotic cells along with 3.32% dead cells were found. In C33A cells, 44.54% cells were found to be apoptotic with 7.52% dead cells. At 24 h treatment time, most of the cells were in the 4^th^ quadrate, with lesser number of early and late apoptotic cells. In HeLa cells, 23.99% cells were found to be apoptotic, with many (41.9%) dead cells. In SiHa cells, most of the cells were dead (82.65%) with 9.02% apoptotic cells. In C33A cells, 77.88% cells were found to be apoptotic with 8.6% dead cells.

Fluorescence micrographs of 18 h and 24 h also support the flow cytometric data (Fig. S3).

#### Change in morphology

**Figure 4:**
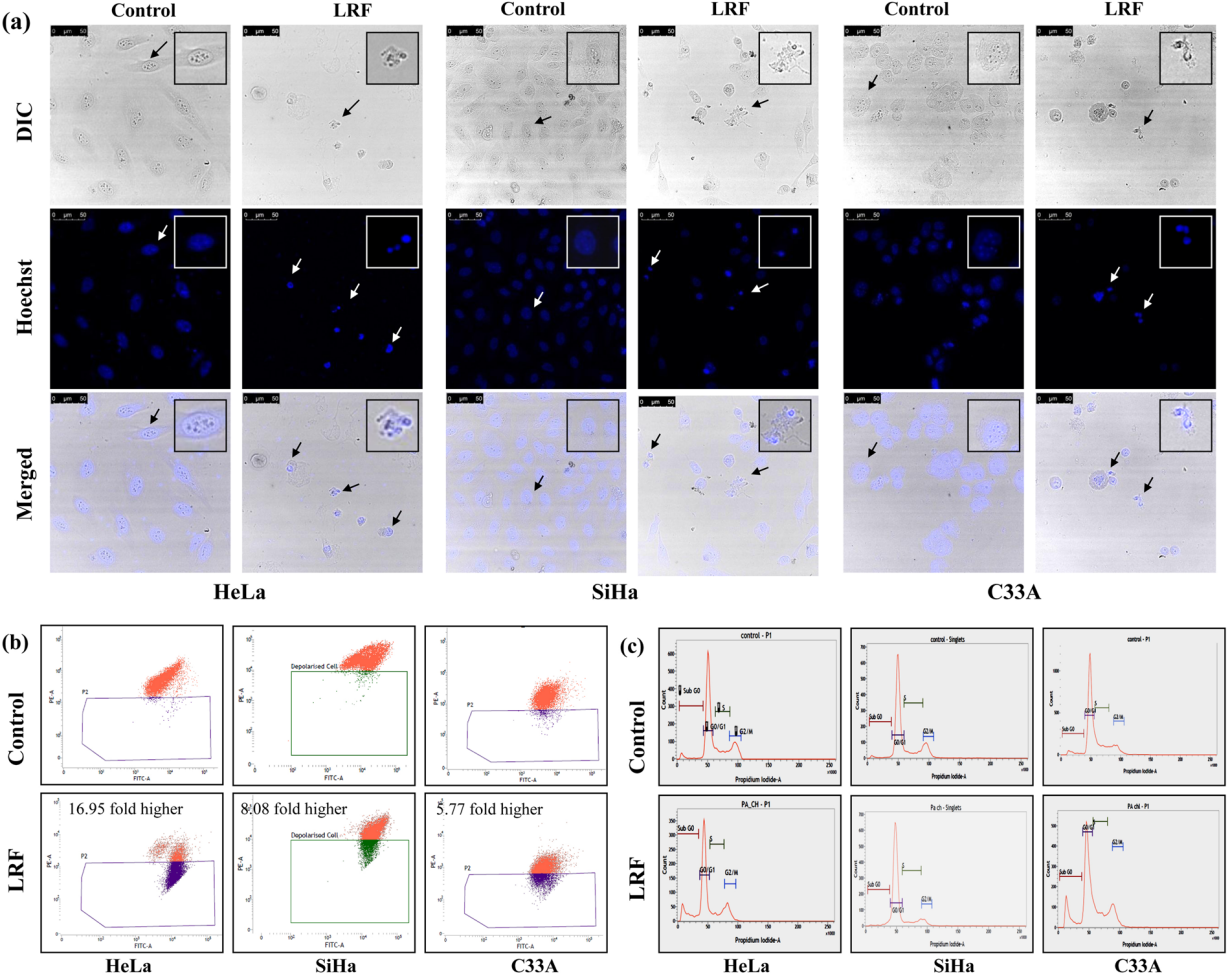
Image and flow cytometric analysis showing apoptosis inducing activity of LRF in cervical cancer cells. (**a**) Representative pictures showing changes in nuclear morphology by LRF treatment. Arrows show apoptotic cells with fragmented/condensed nuclei and membrane blebbings. Scale represents 50 μm. Inset showing magnified pointers. (**b**) Scatter plots showing decrease in MMP in LRF treated cells after 6 h of LRF treatment. (**c**) Histograms showing changes in cell cycle patterns of LRF treated cells at 24 h.

Treated cells showed significant changes in cellular and nuclear morphology as seen by confocal microscopy. In HeLa cells, cytoplasmic blebbings, shrinkage of membrane, chromatin condensation and nuclear fragment, all the signs of typical apoptotic cells were observed (Fig. 4a). In SiHa cells also, cell shrinkage along with chromatin condensation and fragmentation were observed (Fig. 4a). In C33A cells, more cellular shrinkage along with nuclear condensation was found in the treated sets (Fig. 4a). Cells were counted and expressed as number of fragmented/ condensed nuclei present per 100 cells in control and LRF treated sets (Fig. S4). It was observed that, in LRF treated Hela, SiHa and C33A cells, maximum numbers of fragmented/condensed nuclei were 54.74%, 46.87% and 34.92% respectively.

### Loss of mitochondrial membrane potential (MMP)

Mitochondria play an important role in inducing cell death; loss of membrane potential (ΔΨm) is a key event in intrinsic cell death. Reduced MMP was quantified in treated cells (Fig. 4b). In HeLa control sets, 3.27% depolarized cells were observed than the treated cells which showed 55.42% depolarized cells, which is significantly (16.95 fold higher) more than the control sets. In SiHa cells, 3.71% depolarized cells were there in control sets while treated sets had 29.97% depolarized cells (8.08 fold increased). In C33A cells, 5.12% and 29.5% cells were found in control and treated sets respectively, showing a 5.77 fold increase in depolarized cell population.

### Change in cell cycle

With LRF treatment, varied responses were observed. In HeLa and C33A cell lines, sub G0 population has increased suggesting cytotoxicity, whereas in SiHa cells, cells were blocked at G1/S phase. A decrease of 8.79% in G0G1 populations along with an increase of 12.03%in sub G0 population was observed in HeLa cells. In C33A cells, increase of 8.68% in sub G0 population along with 9.48% decrease in G0G1, 3.9% decrease in S and 4.36% increase in G2/M population were observed in treated sets. In SiHa cells, S and G2/M populations decreased by 3.11% and 4.87% respectively while sub G0 population increased by 1.58% in treated sets (Fig. 4c).

### Interplay of cell survival – cell death genes at transcriptional and translational level

#### Semi-Q RT-PCR

In all the treated cells, elevated levels of pro-apoptotic gene and reduced levels of anti-apoptotic gene expressions were observed. Viral HPV 16/18 E6 gene expression was found to be decreased in SiHa and HeLa cells along with the decrease in expression of Endothelin 1(ET-1).

**Figure 5:**
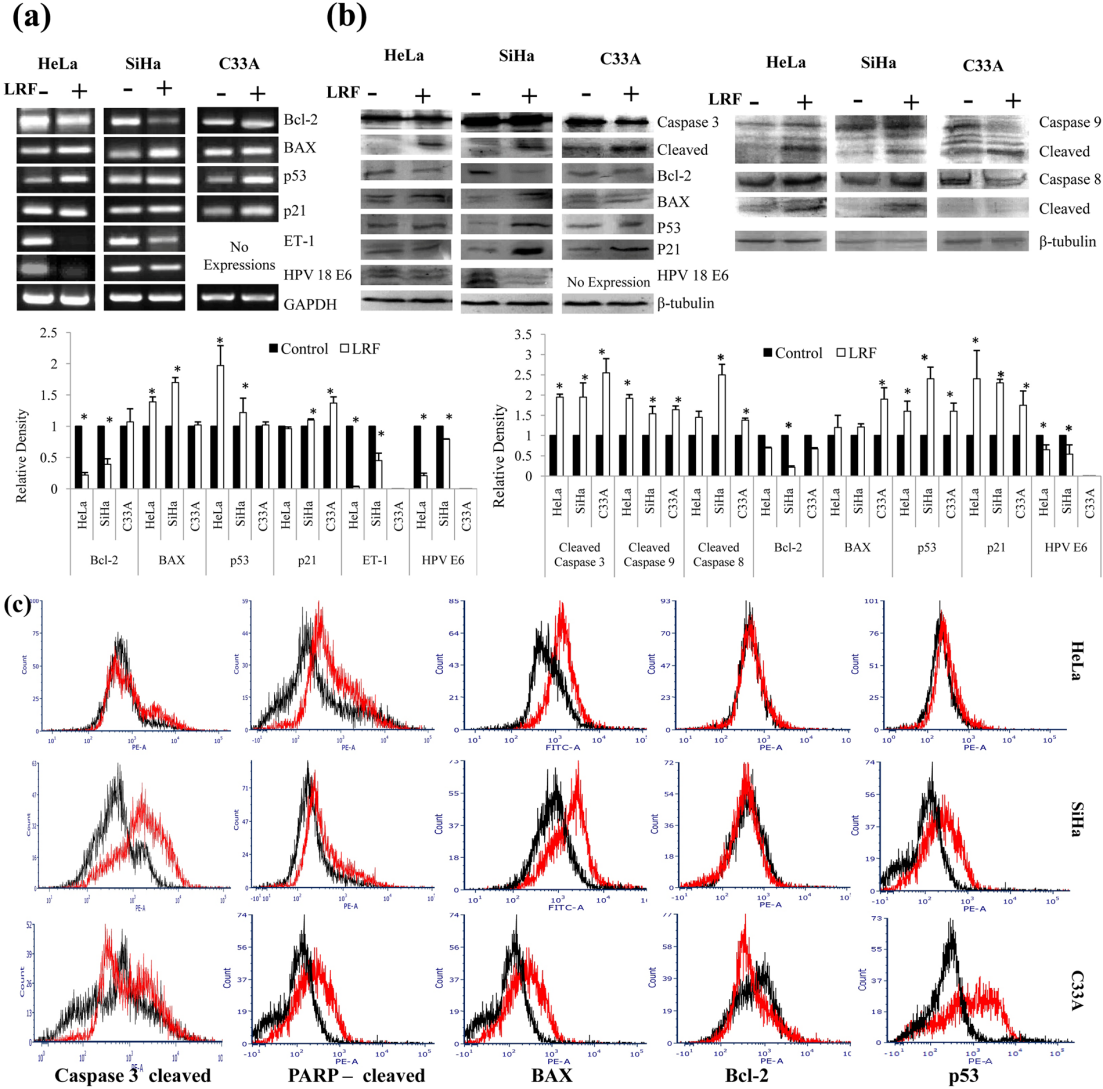
Expression analysis of apoptotic genes and proteins at 24 h LRF treatment. (**a**) Semi Q RT-PCR (**b**) Immunoblotting. Caspase 8 and 9 were studied at 18 h, rest were studied at 24 h. For both experiments, representative figures with fold changes (densitometry) were expressed as histograms. Columns represent the average of relative densities while bars represent standard deviations. *Denotes significant difference between control and treated sets (P < 0.05). [Gel figures for control and treated sets in (**a**) were cropped from same gel. Detailed information was given as Supplementary Information]. (**c**) Representative histogram overlaps showing change in protein expressions using intra cellular flow cytometry. Black and Red lines indicate control and LRF treated sets respectively.

In treated HeLa cells (Fig. 5a), Bcl-2 expression level was decreased (by 0.78 fold) while BAX expression was elevated (by 0.39 fold). p53 expression increased to 0.97 fold higher but decreased expression of p21 was observed (0.04 fold) in the treated sets. Huge decrease in expressions of ET-1(0.97 fold) and HPV 18E6 (0.79 fold) were observed in the treated sets. Treated SiHa cells (Fig. 5a) showed 0.61fold decrease in Bcl-2 expression along with 0.7 fold increase in BAX expression. 0.22 fold increase in p53 expression along with mild increase of 0.1 fold for p21 were observed. Expression of ET-1 and HPV 16 E6 were found to be decreased by 0.55 fold and 0.21 fold respectively. No significant change in expressions of Bcl-2 and BAX was found in treated sets of C33A cells (Fig. 5a). For both the p53 and p21, there was an increase in expressions (0.07 fold and 0.36 fold). ET-1 expression was not detected as expected in this cell line, which didn’t have HPV viral load.

#### Immunoblotting

Balance between the pro-apoptotic and anti-apoptotic proteins decide the cell fate, if proapoptotic proteins expressed more than the antiapoptotic proteins, then the cell dies and *vice-versa*. In the present work, expressions of anti-apoptotic, pro-apoptotic and some relevant proteins were studied by immunoblotting to understand the cell death mechanism. Expression of caspase 8 and 9 were studied at 18 h, while other proteins were studied at 24 h post treatment. All the treated sets showed elevated levels of initiator caspase 8 and 9, effecter caspase3, pro-apoptotic BAX, p53 and reduced levels of anti-apoptotic Bcl-2 proteins. In treated HeLa cells (Fig. 5b), expression of cleaved caspase 3, caspase 9 and caspase 8 were found to be increased by 0.95, 0.92 and 0.45 fold respectively. Decrease in expression of 0.35 fold for Bcl-2 with an increase of 0.2 fold in case of BAX was found. 0.6 fold increase for p53, 1.4 fold increase in case of p21 and 0.48 fold decrease in case of HPV 18 E6 were observed. In HeLa cells expression of cleaved caspase 3,9,8; p53; HPV18 E6 changed significantly. In treated SiHa cells (Fig. 5b) cleaved caspases 3 showed 0.95 fold increase, whereas, 0.54 and1.5 fold increase were observed for caspase 9 and 8, along with 1.1 fold and 1.3 fold increase in expressions of p53 and p21respectively. Expression of Bcl-2 was decreased by 0.86 fold with 0.21 fold increase in BAX expression. Reduced expression of HPV16 E6 was also observed (0.68 fold). In SiHa cells the change in expression of all the proteins studied here showed significant difference with the untreated counter parts (except BAX). In C33A cells (Fig. 5b) 1.25, 0.64 and 0.38 fold increase in cleaved caspases 3,9 and 8 were found along with increase of 0.6 fold and 0.8 fold in p53 and p21 expressions. Reduced expression of Bcl-2 was found (by 0.31 fold) while BAX expression increased by 0.9 fold.Other than Bcl-2, change in all other protein expression is statistically significant in C33A cells.

### Intracellular flow cytometry (ICFC)

#### Cleaved Caspase 3

Increase in cells having cleaved caspase 3 was found in all the treated sets (Fig. 5c). In control HeLa cells, 11.27% cells expressed cleaved caspase 3 while 22.88% cells were found positive for cleaved caspase 3 in treated sets. An increase of 2.03 fold of cells expressing cleaved caspase 3 was detected. In case of SiHa cells, 13.91% cells expressed cleaved caspase 3 in the control set and52.86% cellsexpressed cleaved caspase 3 in the treated sets with an increase of 3.8 fold. 24.77% and 45.36% cells expressed cleaved caspase 3 in control and treated sets respectively in C33A cells with an increase of 1.83 fold.

#### Cleaved PARP

Cells having cleaved PARP increased in all the treated sets (Fig. 5c), highest in HeLa followed by C33A and SiHa cells. In HeLa control sets, 12.02% cells expressed cleaved PARP while 39.09% cells of treated sets expressed cleaved PARP, with an increase of 3.3 folds. In SiHa cells, cells expressing cleaved PARP were 8.34% and 22.63% in control and treated sets respectively with an increase of 2.72 fold. Highest fold increase in cleaved PARP positive cells (3.84 fold) was found in C33A cells with 9.02% cells in control sets and 34.68% in treated sets.

#### BAX

In the treated sets, mean fluorescence intensity of BAX positive cells increased in all the cell lines (Fig. 5c). Maximum expression was observed mostly in C33A cells followed by HeLa and SiHa cells. In HeLa cells, BAX expression increased by 1.75 fold while in SiHa it increased by 1.64 fold. It increases 2.43 fold in C33A.

#### Bcl-2

In the treated sets, mean fluorescence intensity of Bcl-2 positive cells decreased in all the cell lines (Fig. 5c), mostly in SiHa followed by HeLa and C33A cells. In HeLa cells, Bcl-2 decreased by 0.16 fold,but in SiHa and C33A it decreased by 0.19 fold and 0.14 fold respectively.

#### p53

In the treated sets, mean fluorescence intensity of p53 positive cells increased in all cell lines, most in SiHa cells followed by C33A and HeLa cells (Fig. 5c). In HeLa cells, p53 increased by 1.44 fold while in SiHa, it increased by 3.12 fold. 2.29 fold increase of p53 was there in C33A.

### *In silico* analysis of phyllanthin binding with p53 and HPV 16/18 E6

**Figure 6:**
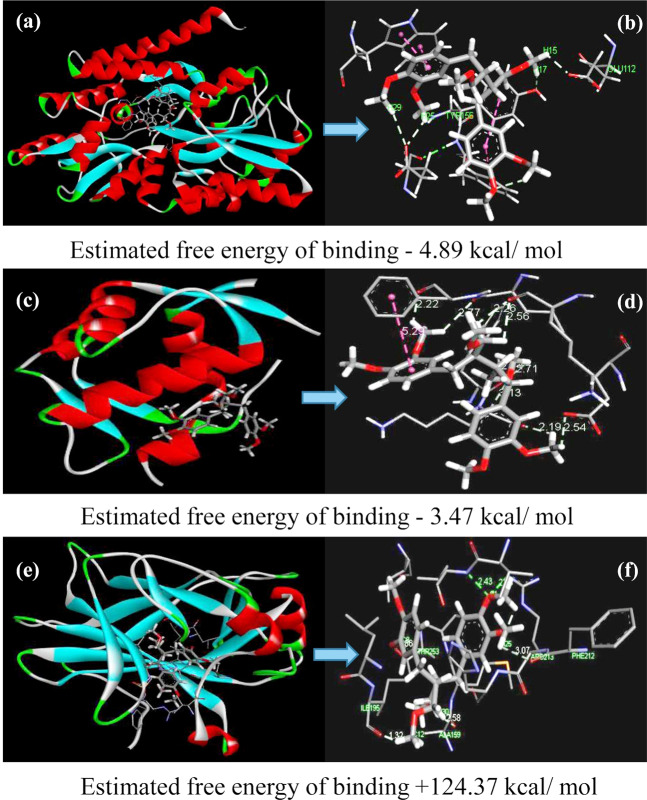
Docked pose showing interaction of (**a**) phyllanthin with E6 protein, (**b**) rear view of phyllanthin with E6 protein, (**c**) phyllanthin with MDM protein, (**d**) rear view of phyllanthin with MDM protein, (**e**) phyllanthin with p53 protein, (**f**) rear view of phyllanthin with p53 protein.

Different types of interactions between phyllanthin and p53, MDM (known inhibitor of p53) and E6 (viral protein which affects p53 stability) were depicted in Fig. 6 and tabulated in Table S3. It was quite clear that phyllanthin had the most rigid binding with E6 protein, having more negative free energy of binding than that of with natural p53 inhibitor MDM2. Interaction between p53 and phyllanthin was not favorable. Table 1 summarizes the hydrogen and pie bond formation by different amino acid residues of the proteins with phyllanthin. Phyllanthin was found to interact with amino acid residue Glu154 and form hydrogen bonds with carbon 7 & 8. It also formed π- Interactions with purine bases and Phe157.

**Table 1:** Dock pose of involving interacting elements of phyllanthin with different amino acid residues of different proteins.

Hydrogen bonds				π- Interactions			
No. of hydrogen bonds	protein End	Compound End	Bond Distances Å	No. of ionic interactions	Compound End	Protein End	Bond Distances Å
**Phyllanthin interacts with E6**							
4	Glu154	H of C-7	2.74	4	Ph ring	2 × purine base	3.60, 4.00
	Glu154	H of C-8	2.18		Another Ph ring	2 × Phe157	4.99, 5.18
**Phyllanthin interacts with MDM**							
8	Phe55	H of C-8 of one phenyl ring	2.22	3	One Ph ring	Phe55	5.29
	Leu54	H of C-8 of one phenyl ring	2.77		One Ph ring	Lys24	4.51
	Lys24	H of C-8 of another phenyl ring	2.19		Another Ph ring	Lys24	4.19
	Glu28	H of C-8 of another phenyl ring	2.54				
	Lys51	2 × H of C-11	2.26, 2.56				
	Trp23	2 × H of C-12	2.71, 2.74				
**Phyllanthin interacts with p53**							
3	Ile195	O-3	2.69	4	One Ph ring	Met160	4.10
	Arg213	O-8	1.86		One Ph ring	Arg213	4.70
	Val173	O-8	1.46		One Ph ring	Val173	4.71
					Another Ph ring	Ile195	4.49

## Discussion

Traditionally, decoction of *Phyllanthus* was used to treat intestinal and liver disorders, but in recent times, anti-diabetic, cardioprotective, anti-inflammatory and anti-cancer properties of this plant (tested on different cell lines) were reported^15^. Along with this, this plant extract was also found to have anti-fungal, anti-bacterial and anti-viral activity^15^.The anti-cancer activity was attributed mainly due to inhibition of cell cycle regulations and DNA repair mechanism^16^. Previous workers have reported that, hexane extract (100 µg/ml) or lignan rich fraction (100 µg/ml) of this plant extract or isolated pure compounds such as niranthin (43 µg/ml), nirtetralin (43.2 µg/ml) and phyllanthin (43 µg/ml) induced cytotoxity on K-562 cells (with 43.3, 66, 62, 61 and 24.2% cell death respectively) but much less cytotoxicity was observed in vincristine resistant Lucena-1 cells (16.3,40.4,29.4, 30.2 and 24.8% respectively). It is interesting to note that K-562 cells are more sensitive (LC50 4.95 µM) to doxorubicin than the resistant Lucena-1 cells, as the LC50 dose was more (LC50 50 µM). But Lucena-1 cells treated with 5 µM Doxorubicin along with *P*. *amarus* derivatives; greater cytotoxicity was observed^17^. Quercetin was reported to inhibit tumor growth and can trigger apoptosis in several types of cancers (cervical cancer, prostate cancer, osteosarcoma and oral cancer)^18–20^. Methanolic extracts of hairy root culture of *P*. *amarus* increased time dependent cytotoxicity in MCF7 cells. That cytotoxicity was found to be associated with elevated levels of ROS as well as decrease in MMP^21^.

DNA damage is very often involved with *in-vitro* cytotoxicity. DNA damage stabilizes p53 concentration, inducing p21 transcription, which is a CDK inhibitor. This p21 then act to arrest the cell cycle at G1 or G2^22^. ROS mediated apoptosis was induced by methanolic extracts of hairy root culture of *P*. *amarus* in MCF7 cells^21^. In our study, it was observed that, short duration treatments (3 h) with IC50 doses generate ROS in the treated cells. ROS induces reduction of mitochondrial membrane potential. ROS generation led to intensive DNA damage and induces DNA damage dependent pathways. DNA damage beyond repair triggers cell death. Apoptosis triggered by various DNA lesions have already been reported by earlier works^23^. LRF was found to induce apoptotic DNA fragmentation, which was evident, as extensive DNA damage was observed both in DNA fragmentation and comet assay (reported in our earlier publication)^24^. So, it can be concluded that LRF treatment induces DNA damage. In the present work, enhanced expressions of proteins related to DNA damage responsive pathways were found. As a result of DNA damage several kinases become active. Out of them, the most important are ATM, ATR and DNA-Pkcs. ATR is activated in single stranded DNA breaks whereas, ATM and DNA-Pkcs are associated with double stranded DNA breaks^25^. As initial response to DNA damage, the histone variant of H2A, H2AX become phosphorylated by ATM/ATR, at the damaged part of the DNA^26,27^. This is essential for accumulation of MDC1 in response to damaged chromatin^28–34^. In our study, western blot analysis showed enhanced expression of phospho-ATR but no expression of phospho-ATM was observed. At 6 h time point expression of phospho-Chk1 increased in treated sets suggesting induction of DNA replication stress^35^. Detection of γ-H2AX also validates the presence of nicks on DNA strands. Expression of γ-H2AX was earlier considered to be associated with double stranded DNA breaks, but recent report showed that, it can also get phosphorylated by ATR dependent manner in response to DNA replicative stress and does not need ATM activation^36^. p21 mediated arrest of cell cycle is also correlated with the phosphorylation of H2AX^37^. Extensive nuclear DNA damage acts as an intrinsic signal to mitochondria, triggering release of cytochrome c to the cytosol and tripping the balance in favour of cell death rather than repair and survival. Loss of membrane potential had led to caspase activation and apoptosis. Quantitative data for ROS generation, loss in MMP and expression of apoptotic proteins with LRF treatment supports this hypothesis.

Hoechst stained cells studied under Confocal microscope, showed clear indication of condensed chromatin, presence of fragmentations was also clear in all the three treated sets indicated apoptotic cell death.

Flow cytometric analysis of theAnnexin V-FITC stained cells at 18 and 24 h after treatment, clearly showed the presence of early apoptotic, late apoptotic and dead cells. At 18 h post treatment, most of the cells were in apoptotic phase, (late apoptotic phase in case of HeLa, C33A and early apoptotic phase in SiHa cell line). But after 24 h, a greater population of cells of HeLa and SiHa were found in the fourth quarter, where as in C33A, the population were in the third quarter. Fluorescence micrographs also supports this observation. DIC pictures, of treated cells showed cytoplasmic blebbings, which are signs of apoptosis are clear enough.

Methanolic extract of *P*. *amarus* was reported to induce caspase-3 mediated apoptosis in DLA cells at a concentration of 75–200 µg/ml^38^. LRF induced cell cycle shifts in the treated cell lines and the degree of response varied among the cell lines. Flow cytometric analysis of cell cycle showed LRF treatment induced G1/S arrest only in SiHa cells, but not in other cell lines (HeLa and C33A cell lines, where increase in sub G0 population was observed). As the cell cycle progress from G1 to S phase, it is evident that cell cycle proteins, that is CDKs and cyclin Ds form an active G1phase MPF that leads the cells from G1 to S. But CKIs (mainly Kip family/ Wnk family inhibitors) may check the progression and arrest the cells in G1/S border, if the cell is not prepared to move into the next phase due to some abnormal conditions (as for example, presence of any DNA damage, etc.). In the present study, it is noted, that p21 have a role to block the progression of cells in G1/S. As p21 blocked the cells at G1/S, lesser number of cells progressed to G2/M.

Caspase 3,7 mediated apoptosis with alternation in MAPK, AKT and NF-kB pathways was reported in PC3 cells by the methanolic extract of *P*. *amarus*^39^. Several signalling pathways are induced simultaneously in the treated sets and p53 acted as the prime regulator that might have controlled these pathways. In general, HPV infected cells; viral E6 binds p53 and disrupts its transcription, thereby impairing its tumor suppressor activity. But, in this study, E6 expression (both transcriptionally and translationally) was found to be reduced significantly. Down regulation of E6 in the treated counter parts of HPV(16/18) positive cells helped in p53 stabilization and subsequent p21 transcription.It was observed, that LRF has induced DNA damages in the cells (as it was evident by incorporation of γ-H2AX in the treated nuclei). Semi qRT-PCR showing the status of ET-1 in treated sets, clearly indicated that, the expression of ET-1 was absent in HeLa cells and down regulated inSiHa cells, both treated with LRF. Endothelin-1 (ET-1) is an autocrine signalling growth factor, released from HPV transfected keratinocytes. It is a vasoactive peptide and induces increased growth response and cell proliferation either directly or in synergy with other growth factors. So, reduced ET-1 expression clearly vindicated that LRF was not only able to induce cell death but also inhibited the cell proliferation of the cancer cell lines. Immunoblotting analysis also showed the enhanced expression of the pro apoptotic proteins over antiapoptotic proteins. Expression of cleaved caspase 8 and 9 (studied at 18 h) increased considerably in the treated cells. As caspase 8 is reported to inhibit autophagic or necrotic cell death^40,41^, it’s activation indicates apoptotic cell death. LRF induced apoptosis was mediated by pro-apoptotic proteins, such as BAX, cleaved PARP and cleaved caspase 3, as, increased expression was detected by intra cellular flow cytometry analysis. At the same time, ROS mediated DNA damage and loss of mitochondrial membrane potential, clearly indicated p53 mediated intrinsic pathway of apoptosis.

The docking results clearly suggest that phyllanthin has more affinity towards E6 than p53. Being a natural inhibitor of p53, MDM2 served as the positive control in this experiment. Figure 6 depicts the interaction of different amino acids of the proteins with phyllanthin. Strong binding capability as well as interactions through hydrogen bonds with different amino acids of E6 made phyllanthin a good lead molecule for E6 inhibition.

**Figure 7:**
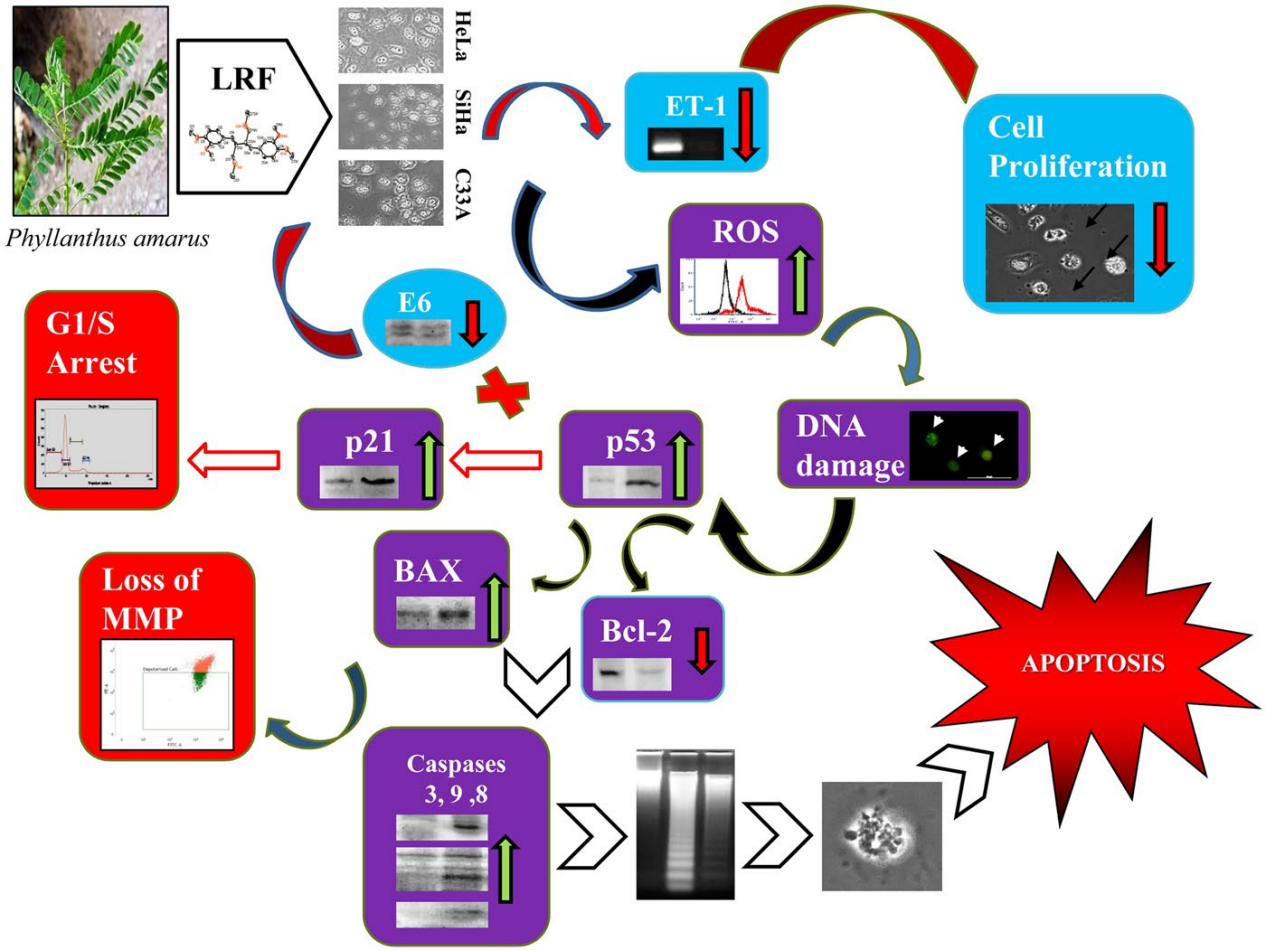
Proposed mode of action of the LRF on the cervical cancer cells. Downward and upward arrows indicate downregulation and upregulation of several genes.

From the study, it can be concluded that LRF has the potential to induce cell death in cervical cancer cells *in vitro*. LRF may had induced cell death through ROS, which lead to DNA damage and loss of MMP. Presence of nicked DNA and subsequent ATR activation pathway also validates the hypothesis. Induction of ATR activation pathway has vindicated the hypothesis. Down regulation of viral E6 is also instrumental in inducing up regulation of p53. p53 played the central role in inducing apoptosis in the cell lines. The presence of viral factors in HeLa and SiHa and their subsequent down regulation by LRF treatment actually made them more susceptible towards this treatment that the C33A cell line which is devoid of any viral load. The probable mode of action of LRF fraction is summarized in Fig. 7. The anti-proliferative activity of LRF might be due to synergistic actions of the components (phyllanthin, fatty acids, niranthin, corrilagin etc.) present in them. Activation of caspase 8, 9 and 3 indicates towards apoptotic cell death.

## Materials and Methods

### Chemicals and reagents

Chemicals needed for chemical elucidation (methanol, n-Hexane, ethyl acetate, chloroform, Silica gel) were purchased from Merck; BSTFA was from Sigma. Cell culture media (EMEM, DMEM), FBS, FCS, antibiotic-antimycotic solutions were procured from HiMedia Labs. DCFDA (ABCAM 113851), JC1 (BD Biosciences551302), AnnexinV-FITC/PI (BD-Biosciences556547) and Propidium iodide (Sigma) were used in the flow cytometric assays. Primers for RT-PCR were from Sigma. RNA extracted by TRI reagent (Sigma) and 1^st^ strand cDNA systhesis kit (Thermo Scientific K1621) was used. Antibodies for WB, IF and ICFC were purchased from Cell signaling technology, Sigma and Santa Cruz biotechnology (Table S4). Tris, Glycine, glacial acetic acid, EDTA and SDS used in gel electrophoresis were from Merck and Invitrogen.

### Plant collection and extraction

*Phyllanthus amarus* plants were collected from the Departmental garden, identified properly, and deposited to Calcutta University Herbarium for accession number (20015-CUH). The whole plants were washed, shade dried and extracted with methanol, concentrated and defatted with n-Hexane. Then it was fractioned with chloroform to get the lignan rich fraction (LRF). Previous studies has helped in determination of IC50 doses in different cell lines^24^. IC50 dose was used to carry out all the experiments at different time points.

### Elucidation of chemicals constitutes

#### LC-MS

The protocol for LCMS was followed according to our previous publication^42^. LRF was dehydrated using gentle stream of nitrogen and further reconstituted in methanol. Then the extract was analyzed by LC-MS/MS (SYNAPT G2 HDMS, Waters, Manchester, U.K). An Acquity UPLC BEH C18 Column (3.0 mm × 150 mm, 1.7 μm, Waters, Ireland) was used for the chromatographic separation at a temperature of 40 °C. Eluent A (0.1% formic acid in water, v/v) and eluent B (0.1% formic acid in acetonitrile, v/v) were the constituents of the mobile phases. At a flow rate of 0.4 mL/min with a linear gradient program these eluents were delivered as follows: 20–80% B from 0 to 15 min, 80–95% B from 15.0 to 15.5 min, held at 95% B from 15.5 to 18.0 min, 95–20% B from 18.0 to 19.0 min and held at 25% B from 19.0 to 20.0 min. The parameters maintained during this operation were as follows: capillary voltage of 3 kV (ESI+), sample cone voltage of 35 V, extraction cone voltage of 4 V, source temperature of 100 °C, desolvation temperature of 300 °C, cone gas flow of 50 L/h and desolvation gas flow of 800 L/h. The trap collision energy in MSE mode, for the low-energy function was set at 5 eV, while the for the high-energy function, ramp trap collision energy was set at 20–50 eV. In MSE mode, for collision-induced dissociation (CID), Argon was used as the collision gas. The mass spectrometer was calibrated over a range of 50–1500 Da using a solution of sodium formate to ensure mass accuracy and reproducibility.

#### GC-MS

LRF was TMS-derivatized with BSTFA and injected in HP5-MS capillary column (30 m X 0.25 mm × 0.25 µm) followed by running through a temperature gradient of 70–260 °C with 5 °C per min ramping rate (Agilent). Fragmentation pattern of the peaks were tallied with NIST library.

#### Column chromatography

LRF was further separated in a silica gel column (60–120 mesh) to get several sub-fractions. Concentrated LRF was mixed with silica gel (60–120) to prepare slurry which was loaded in a glass column packed with silica gel (60–120).Elution was done with hexane, ethyl acetate and methanol according to increasing order of polarity. Sub-fractions were collected for ½ of the column bed volume. They were then concentrated and separated by thin layer chromatography followed by poolingwhen necessary. Pooled fractions were tested for their cytotoxicity and analysed by GC-MS.

#### Characterization of single crystal

The crystal was mounted (Bruker AXS D8 Quest ECO) with the help of nylon looponthe diffractometer. The diffractometer contained a Mo-target rotating-anode X-ray source and a graphite monochromator (Mo Kα, λ = 0.71072 Ǻ). The crystallographic data have been listed in Table S2. SHELXS-97 (Sheldrick 2008) and SHELXL-2014/6 (Sheldrick 2014) were used for data solution and data refinement respectively. The crystal data was submitted to CCDC. A Bruker AM 300 L 500 MHz superconducting FT NMR spectrophotometer was used to record the ^1^H NMR spectra for the isolated crystal resolved in deuterated chloroform (CDCl3) at 293 K. MicromassQtof YA 263 mass spectrometer was used to measure the ESI mass spectra in positive ion mode.

### ROS induction by flow cytometry

1 × 10^6^ cells were stained with DCFDA following manufacturer’s protocol and analysed by flow cytometry (BD FACSVerse). In presence of ROS, DCFDA, a cell permeant fluorogenic dye readily oxidizes to 2,7-Dichlorofluorescin which is highly fluorescent.

### Immunofluorescence microscopy to detect DNA damage

To detect DNA damages, after treatment 1 × 10^4^cells were fixed with 4% PFA, stained with fluorescein isothiocyanate (FITC) conjugated (1:100) anti γ-H2AX antibody (1:50) overnight, (Table S4) and photographed under fluorescent microscope (Leica).

### Determination of loss of mitochondrial membrane potential (MMP) by flow cytometry

After treatment, 1 × 10^6^ cells were stained with JC1 following manufacturer’s protocol and analysed by flow cytometry (BD FACSVerse) to study change in the MMP. JC1 is a lipophilic cationic dye that can selectively enter mitochondria. With intact MMP, it exists as aggregates. It’sfluorescence changes from red (aggregates) to green (monomers) with decrease in MMP.

### Determination of apoptosis by annexin V-FITC/ PI double staining

FITC conjugated Annexin V/ Propidium Iodide double staining method was used to quantifyamount of apoptotic cells. Externalization of phosphatidylserine (PS) is a well known marker of apoptosis. Annexins, a family of calcium-dependent phospholipid binding proteins, bind to PS and helps to mark apoptotic cells. After treatment, 1 × 10^6^ cells were harvested, washed with PBS, stained with FITC conjugated Annexin V/Propidium Iodide for 15 min at dark according to manufacturer’s protocol (BD 556547). Later they were subjected to flow cytometry (BD Accuri C6 plus/ BD FACSVerse) and analysed by FCS-express software. Also cells were grown on glass cover slips and treated with LRF for 18 and 24 h, stained with Annexin V-FITC/ PI and visualized under epi-fluroscence microscope (Leica DMi8).

### Cellular and nuclear morphology study. 

Hoechst 33258 stained cells were studied under fluorescent microscope. Then the treated cells were washed with PBS and fixed with 4% PFA followed by washing again. The fixed cells were then stained using Hoechst 33258 (2 µg/ml) for 2 min at room temperature. They were mounted in mounting medium (PBS:Glycerol = 1:9) containing propyl gallate after washing with PBS. Photographs were taken in an confocal microscope (Leica DMi8).

Cell cycle analysis Treated cells were harvested and washed with PBS. 70% ethanol was used to fix the cells which were then treated with RNaseA for 2 h at 37 °C and stained with PI. After 15 min incubation in dark, flowcytometry (BD FACSVerse) was done to study cell cycle.

### Semi quantitative reverse transcription PCR

Tri regent was used to isolate total RNA from the cells as depicted from the manufacturer’s protocol. RNA was quantified by Nanodrop (Eppendorf) and bleach gels were used to check the integrity of the RNA samples^43^. First strand cDNA synthesis kit was employed to prepare cDNA using 1 µg RNA as starting material as per manufacturer’s protocol. With the help of Primer 3 software,primersrequired for the PCR reactions were designed, using FASTA sequences of target genes from NCBI. The primer sequences and PCR profiles were listed in (Table S5).GAPDH was used as internal control. PCR products were separated in 2% Agarose gels and Gel documentation system (UVP MultiDoc-It) was used to photograph them. Densitometric analysis was done by Image J software.

### Immunoblotting

Total protein was extracted with Laemmli’s SLB (sample loading buffer) (50 mMTris-Cl pH 6.8, 0.01% Bromophenol blue,10% 2-Mercaptoethanol, 10% Glycerol, 2% SDS, protease and phosphatase inhibitors), quantified. 50–100 µg of total protein was separated on polyacrylamide gels of required percentage.Proteins were blotted on a nitrocellulose membrane (Pall-0.22 µm). 5% BSA was used as a blocking solution for 3 h at room temperature. Then they were incubated with primary and secondary antibodies respectively (Table S4). Blots were developed with NBT-BCIP and photographed in gel doc (UVP) and densitometric analysis was done by Image J software.

### Intracellular flow cytometry

For ICFC, 1 × 10^6^ fixed (4% PFA) cells were stained with appropriate dilutions of fluorochrome conjugated antibodies (Table S4) according to manufacturers’ protocol and analysed by flow cytometry (BD FACSVerse).

### *In silico* analysis of phyllanthin binding with p53 and HPV 16/18 E6

Docking was performed with DFT optimized geometries of phyllanthin. For docking studies the structural coordinates of E6, MDM and p53were fetched from the protein data bank (PDB) ID: 4giz^44^, 1ycr^45^ and 1tup^46^ respectively. AutoDock 4.2^47^ was used to carry out molecular docking for the compound. Keeping the conformations of the protein fixed, conformations of the compound were allowed to change during the docking. The data obtained from docking was examined by PyMOL software^48^. Discovery Studio 4.1 Client and Chimera 1.10.1rc were used for visualization effects.

### Statistical analysis

All experiments were performed in triplicates. Averages and standard deviations were calculated by Microsoft Excel. One way ANOVA followed by Duncan’s multiple comparison test was done by GraphPad Prism 5 software.

## Supplementary information

Supplementary info

## References

[1] Bray, F. *et al*. Global Cancer Statistics 2018: GLOBOCAN Estimates of Incidence and Mortality Worldwide for 36 Cancers in 185 Countries. *CA Cancer J Clin.* **68**, 394–424 (2018). 30207593 10.3322/caac.21492

[2] Thomas, M., Pim, D. & Banks, L. The role of the E6-p53 interaction in the molecular pathogenesis of HPV. *Oncogene.* **18**, 53 (1999). 10.1038/sj.onc.1202953 10618709

[3] Rosanò, L., Spinella, F. & Bagnato, A. Endothelin 1 in cancer: biological implications and therapeutic opportunities. *Nature reviews. Cancer.* **13**(9), 637 (2013). 23884378 10.1038/nrc3546

[4] Ehrke, M. J. Immunomodulation in cancer therapeutics. *International immunopharmacology.* **3**(8), 1105–1119 (2003). 12860167 10.1016/S1567-5769(03)00021-3

[5] Gottesman, M. M. Mechanisms of cancer drug resistance. *Annual review of medicine.* **53**(1), 615–627 (2002). 11818492 10.1146/annurev.med.53.082901.103929

[6] Gurib-Fakim, A. Medicinal plants: traditions of yesterday and drugs of tomorrow. *Molecular aspects of Medicine.* **27**(1), 1–93 (2006). 16105678 10.1016/j.mam.2005.07.008

[7] National Medicinal Plants Board, (www.nmpb.nic.in).

[8] Calixto, J. B., Santos, A. R., Cechinel, V. F. & Yunes, R. A. A review of the plants of the genus *Phyllanthus*: their chemistry, pharmacology, and therapeutic potential. *Med. Res. Rev.* **18**(4), 225–258 (1998). 9664291 10.1002/(sici)1098-1128(199807)18:4<225::aid-med2>3.0.co;2-x

[9] Santos, A. R. S., Ailho, V. C., Yunes, R. A. & Calixto, J. B. Analysis of the mechanism underlying the Anti-nociceptive Effect of the Extracts of plants from the Genus *Phyllanthus*. *General Pharmacol.* **26**, 1499–1506 (1995). 10.1016/0306-3623(95)00030-5 8690236

[10] Ezzat, S. M. *et al*. Anticancer potentiality of lignan rich fraction of six Flaxseed cultivars. *Sci. Rep.* **8**, 544 (2018). 29323210 10.1038/s41598-017-18944-0 PMC5764973

[11] Gordaliza, M., Castro, M. A., del Corral, J. M. & Feliciano, A. S. Antitumor properties of podophyllotoxin and related compounds. *Curr Pharm Des.* **6**, 1811–1839 (2000). 11102564 10.2174/1381612003398582

[12] Jia, L. *et al*. A potential anti-tumor herbal medicine, Corilagin, inhibits ovarian cancer cell growth through blocking the TGFβ signaling pathways. *BMC Complement Altern Med* **13**, 33 (2013). 23410205 10.1186/1472-6882-13-33 PMC3598193

[13] Chen, H. *et al*. Anti-Tumor Effect of Rutin on Human Neuroblastoma Cell Lines through Inducing G2/M Cell Cycle Arrest and Promoting Apoptosis. *The Scientific World Journal*. **2013**, Article ID 269165, 8 (2013). 10.1155/2013/269165 PMC3891239 24459422

[14] Hashemza, M. *et al*. Anticancer and apoptosis inducing effects of quercetin *in vitro* and *in vivo*. *Oncology Reports* **38**, 819–828 (2017). 28677813 10.3892/or.2017.5766 PMC5561933

[15] Meena, J., Sharma, R. A. & Rolania, R. A review on phytochemical and pharmacological properties of *Phyllanthus amarus*Schum. and Thonn. *International journal of Pharmaceutical sciences and Research* **9**(4), 1377–1386 (2018).

[16] Rajeshkumar, N. V. *et al*. Antitumour and anticarcinogenic activity of *Phyllanthus amarus* extract. *Journal of Ethnopharmacology* **81**(1), 17–22 (2002). 12020923 10.1016/s0378-8741(01)00419-6

[17] Leite, D. F. *et al*. The cytotoxic effect and the multidrug resistance reversing action of lignans from *Phyllanthus amarus*. *Plantamedica* **72**(15), 1353–1358 (2006). 10.1055/s-2006-951708 17054045

[18] Sak, K. Site-specific anticancer effects of dietary flavonoid quercetin. *Nutr. Cancer* **66**, 177–193 (2014). 24377461 10.1080/01635581.2014.864418

[19] Bishayee, K. *et al*. Quercetin induces cytochrome-c release and ROS accumulation to promote apoptosis and arrest the cell cycle in G2/M, in cervical carcinoma: signal cascade and drug-DNA interaction. *Cell Prolif* **46**, 153–163 (2013). 23510470 10.1111/cpr.12017 PMC6495502

[20] Gokbulut, A. A., Apohan, E. & Baran, Y. Resveratrol and quercetin-induced apoptosis of human 232B4 chronic lymphocytic leukemia cells by activation of caspase-3 and cell cycle arrest. *Hematology* **18**, 144–150 (2013). 23432965 10.1179/1607845412Y.0000000042

[21] Abhyankar, G. *et al*. Hairy root extract of *Phyllanthus amarus* induces apoptotic cell death in human breast cancer cells. *Innovative food science & emerging technologies* **11**(3), 526–532 (2010).

[22] Jimeno, A. *et al*. Pharmacodynamic-guided modified continuous reassessment method–based, dose-finding study of rapamycin in adult patients with solid tumors. *Journal of clinical oncology* **26**(25), 4172–4179 (2008). 18757332 10.1200/JCO.2008.16.2347 PMC2654371

[23] Roos, W. P. & Kaina, B. DNA damage-induced cell death by apoptosis. *Trends Mol Med* **12**, 440–450 (2006). 16899408 10.1016/j.molmed.2006.07.007

[24] Paul, S., Chakraborty, S., Mukherjee, A. & Kundu, R. Evaluation of Cytotoxicity and DNA Damaging Activity of Three Plant Extracts on Cervical Cancer Cell Lines. *International Journal of Pharmaceutical Sciences Review and Research* **31**(37), 183–189 (2015).

[25] Durocher, D. & Jackson, S. P. DNA-PK, ATM and ATR as sensors of DNA damage: variations on a theme? *Current opinion in cell biology* **13**(2), 225–231 (2001). 11248557 10.1016/s0955-0674(00)00201-5

[26] Zhang, D., Zaugg, K., Mak, T. W. & Elledge, S. J. A role for the deubiquitinating enzyme USP28 in control of the DNA-damage response. *Cell* **126**(3), 529–542 (2006). 16901786 10.1016/j.cell.2006.06.039

[27] Dong, Y. *et al*. Regulation of BRCC, a holoenzyme complex containing BRCA1 and BRCA2, by a signalosome-like subunit and its role in DNA repair. *Molecular cell* **12**(5), 1087–1099 (2003). 14636569 10.1016/s1097-2765(03)00424-6

[28] Nijman, S. M. *et al*. The deubiquitinating enzyme USP1 regulates the Fanconianemia pathway. *Molecular cell* **17**(3), 331–339 (2005). 15694335 10.1016/j.molcel.2005.01.008

[29] Huang, T. T. *et al*. Regulation of monoubiquitinated PCNA by DUB autocleavage. *Nature cell biology* **8**(4), 341 (2006). 10.1038/ncb1378 16531995

[30] Paull, T. T. *et al*. A critical role for histone H2AX in recruitment of repair factors to nuclear foci after DNA damage. *Current Biology* **10**(15), 886–895 (2000). 10959836 10.1016/s0960-9822(00)00610-2

[31] Celeste, A. *et al*. H2AX haploinsufficiency modifies genomic stability and tumor susceptibility. *Cell* **114**(3), 371–383 (2003). 12914701 10.1016/s0092-8674(03)00567-1 PMC4737479

[32] Celeste, A. *et al*. Histone H2AX phosphorylation is dispensable for the initial recognition of DNA breaks. *Nature cell biology* **5**(7), 675 (2003). 12792649 10.1038/ncb1004

[33] Stucki, M. *et al*. MDC1 directly binds phosphorylated histone H2AX to regulate cellular responses to DNA double-strand breaks. *Cell* **123**(7), 1213–1226 (2005). 16377563 10.1016/j.cell.2005.09.038

[34] Lou, Z., Minter-Dykhouse, K., Wu, X. & Chen, J. MDC1 is coupled to activated CHK2 in mammalian DNA damage response pathways. *Nature* **421**(6926), 957 (2003). 12607004 10.1038/nature01447

[35] Gupta, D., Lin, B., Cowan, A. & Heinen, C. D. ATR-Chk1 activation mitigates replication stress caused by mismatch repair-dependent processing of DNA damage. *Proc Natl Acad Sci USA* **115**(7), 1523–1528 (2018). 29378956 10.1073/pnas.1720355115 PMC5816205

[36] Ward, M. I. & Chen, J. Histone H2AX Is Phosphorylated in an ATR-dependent Manner in Response to Replicational Stress. *The Journal of Biological Chemistry* **276**(51), 47759–47762 (2001). 11673449 10.1074/jbc.C100569200

[37] Fragkos, M., Jurvansuu, J. & Beard, P. H2AX Is Required for Cell Cycle Arrest via the p53/p21 Pathway. *Molecular and Cellular Biology* **29**(10), 2828–2840 (2009). 19273588 10.1128/MCB.01830-08 PMC2682023

[38] Harikumar, K. B., Kuttan, G. & Kuttan, R. *Phyllanthus amarus* inhibits cell growth and induces apoptosis in Dalton’s lymphoma ascites cells through activation of caspase-3 and downregulation of Bcl-2. *Integrative cancer therapies* **8**(2), 190–194 (2009). 19223368 10.1177/1534735408330713

[39] Tang, Y. Q., Jaganath, I. B. & Sekaran, S. D. *Phyllanthus* spp. induces selective growth inhibition of PC-3 and MeWo human cancer cells through modulation of cell cycle and induction of apoptosis. *PLoS One* **5**(9), e12644 (2010). 20838625 10.1371/journal.pone.0012644 PMC2935893

[40] Holler, N. *et al*. Fas triggers an alternative, caspase-8–independent cell death pathway using the kinase RIP as effector molecule. *Nature immunology* **1**(6), 489 (2000). 11101870 10.1038/82732

[41] Hou, W., Han, J., Lu, C., Goldstein, L. A. & Rabinowich, H. Autophagic degradation of active caspase-8: a crosstalk mechanism between autophagy and apoptosis. *Autophagy* **6**(7), 891–900 (2010). 20724831 10.4161/auto.6.7.13038 PMC3039736

[42] Paul, S. & Kundu, R. ROS mediated DNA damage and induction of apoptosis in cervical cancer cells by *Heliotropium indicum* L. *Journal of Applied Pharmaceutical Science* **8**(08), 92–106 (2018).

[43] Aranda, P. S., LaJoie, D. M. & Jorcyk, C. L. Bleach gel: a simple agarose gel for analyzing RNA quality. *Electrophoresis* **33**(2), 366–369 (2012). 22222980 10.1002/elps.201100335 PMC3699176

[44] Zanier, K. *et al*. Structural basis for hijacking of cellular LxxLL motifs by papillomavirus E6 oncoproteins. *Science* **339**, 694–698 (2013). 23393263 10.1126/science.1229934 PMC3899395

[45] Kussie, P. H. *et al*. Structure of the MDM2 oncoprotein bound to the p53 tumor suppressor transactivation domain. *Science* **274**, 948–953 (1996). 8875929 10.1126/science.274.5289.948

[46] Cho, Y., Gorina, S., Jeffrey, P. D. & Pavletich, N. P. Crystal structure of a p53 tumor suppressor-DNA complex: understanding tumorigenic mutations. *Science.* **265**, 346–355 (1994). 8023157 10.1126/science.8023157

[47] Morris, G. M. *et al*. AutoDock4 and AutoDockTools4: Automated docking with selective receptor flexibility. *Journal of computational chemistry.* **30**, 2785–2791 (2009). 19399780 10.1002/jcc.21256 PMC2760638

[48] DeLano, W. L. PyMOL: An Open-Source Molecular Graphics Tool, http://pymol.sourceforget.net/ (2002).

