# Author Correction: Lignan enriched fraction (LRF) of *Phyllanthus amarus* promotes apoptotic cell death in human cervical cancer cells *in vitro*

**DOI:** 10.1038/s41598-025-87071-y

**Published:** 2025-02-11

**Authors:** Subhabrata Paul, Debashis Patra, Rita Kundu

**Affiliations:** 1 Bioprospecting Laboratory, School of Biotechnology, Department of Life Sciences, Presidency University, Kolkata, India; 2 Chemistry Laboratory, Department of Science & Humanities, Acharya Jagadish Chandra Bose Polytechnic, Berachampa, West Bengal India; 3 Cell Biology Laboratory, Department of Botany, Centre of Advanced Studies, University of Calcutta, Kolkata, India

Correction to: *Scientific Reports* 10.1038/s41598-019-51480-7, published online 18 October 2019

The original Article contains errors. Due to an error during figure assembly, in Figure 3B the LRF (24 h) panel of C33A cells was invertedly duplicated from Fig 6B in [1] (reference 42 in the original article). The experiments shown in this Article and [1] have been performed at the same time.

The corrected Figure 3 appears below as Figure 1:

**Fig. 1:**
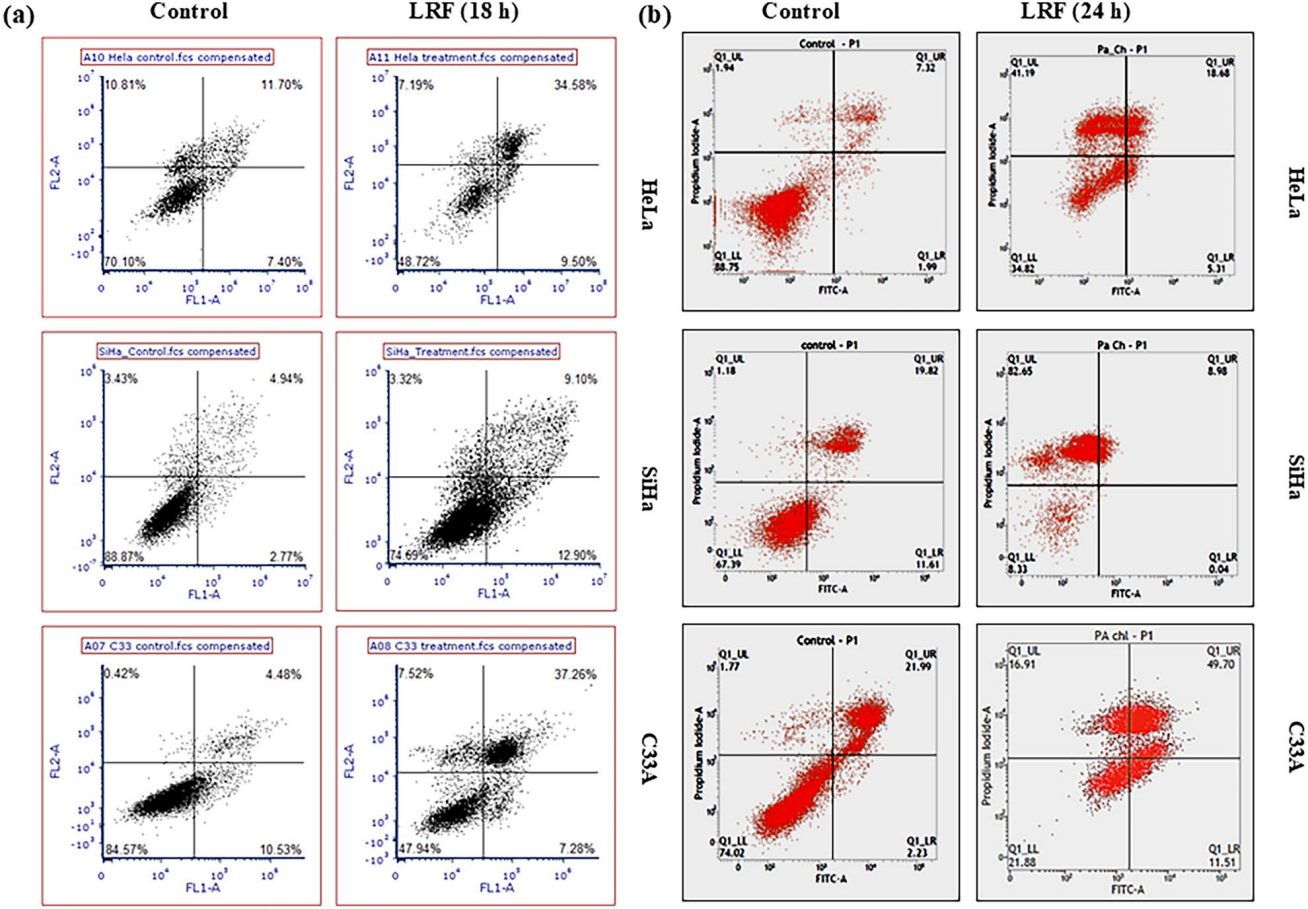
Flow cytometric analysis showing apoptosis inducing activity of LRF in cervical cancer cells at 18 h (**a**) and 24 h (**b**) in Hela, SiHa and C33A cells respectively; in section (**a**) X axis (FL1-A) corresponds to FITC and Y axis (FL2-A) corresponds to PI.

As a consequence, in the Results section under the subheading ‘Determination of Apoptosis inducing potential of LRF’ the sentence:

In C33A cells, 77.88% cells were found to be apoptotic with 8.6% dead cells.

should read:

In C33A cells, 61.21% cells were found to be apoptotic with 16.91% dead cells.

In addition, due to errors during figure assembly, the qPCR bands in Figure 5A Hela plus LRF Bcl-2, p53, p21, and E6 are the same as Fig 10 lane 2 in [1] and in Fig 5C the C33A Bax and Parp-cleaved FACS profiles show the same image as SiHa p53 profile. During the preparation of this correction, the Authors noticed that the quantification of Fig 5A C33A p53 and the error bars in the quantification of p21 are incorrect.

The corrected Figure 5 appears below as Figure 2:

**Fig. 2:**
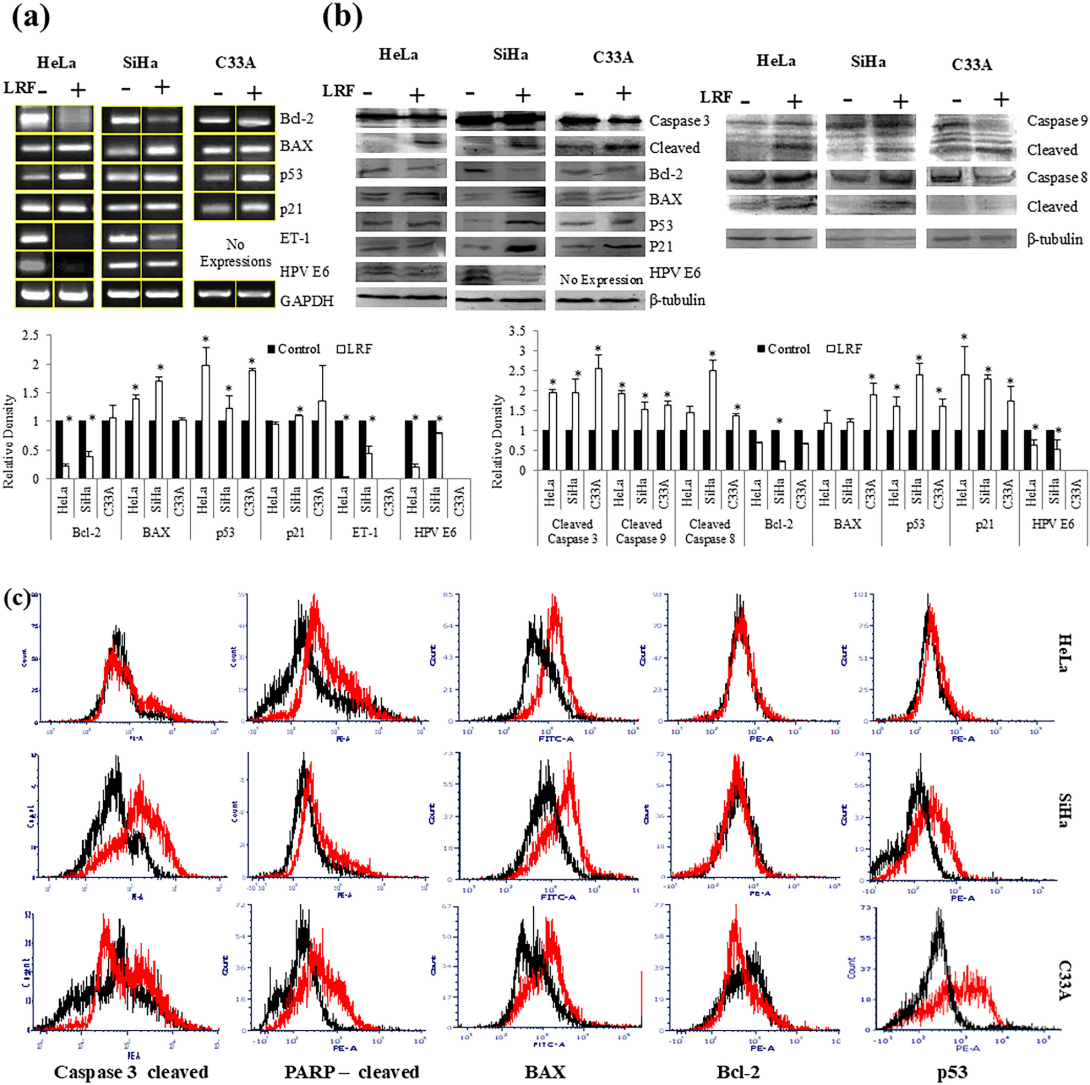
Expression analysis of apoptotic genes and proteins at 24 h LRF treatment. (**a**) Semi Q RT-PCR (**b**) Immunoblotting. Caspase 8 and 9 were studied at 18 h, rest were studied at 24 h. For both experiments, representative figures with fold changes (densitometry) were expressed as histograms. Columns represent the average of relative densities while bars represent standard deviations. *Denotes significant difference between control and treated sets (P < 0.05). [Gel figures for control and treated sets in (**a**) were cropped from same gel. Detailed information was given as Supplementary Information]. (**c**) Representative histogram overlaps showing change in protein expressions using intra cellular flow cytometry. Black and Red lines indicate control and LRF treated sets respectively.

Consequently, in the Results section under the subheading ‘Interplay of cell survival – cell death genes at transcriptional and translational level’ the sentence:

For both the p53 and p21, there was an increase in expressions (0.07 fold and 0.36 fold).

should read:

For both the p53 and p21, there was an increase in expressions (0.88 fold and 0.36 fold).

The same control conditions as in [1] have been used in the following figures and panels:

- Fig 2B Hela Control panels are the same as the control panels in Fig 22 A, B, C in [1];
- Fig 2B SiHa Control panels are the same as the control panels in Fig 23 A, B, C in [1];
- Fig 2B C33A Control panels are the same as the control panels in Fig 24 A, B, C in [1];
- Fig 3B Hela Control panel is the same as Fig 4A in [1];
- Fig 3B SiHa Control panel is the same as Fig 5A in [1];
- Fig 3B C33A Control panel is the same as Fig 6A in [1];
- Fig 4C Hela Control panel is the same as Fig 7A in [1];
- Fig 4C SiHa Control panel is the same as Fig 8A in [1];
- Fig 4C C33A Control panel is the same as Fig 9A in [1];
- Fig 5A Hela minus LRF is the same as Fig 10 lane 1 in [1];
- Fig 5A SiHa minus LRF is the same as Fig 11 lane 1 in [1];
- Fig 5A C33A minus LRF is the same as Fig 12 lane 1 in [1].

[1] Paul, S. & Kundu, R. ROS mediated DNA damage and induction of apoptosis in cervical cancer cells by *Heliotropium indicum* L. *Journal of Applied Pharmaceutical Science* **8**(08), 92–106 (2018).

