# Supplementary material for: Lignan enriched fraction (LRF) of Phyllanthus amarus promotes apoptotic cell death in human cervical cancer cells in vitro
Source: Sci Rep. 2019 Oct 18;9:14950. doi: 10.1038/s41598-019-51480-7 (PMC6802087; doi:10.1038/s41598-019-51480-7)
Supplement: Supplementary file 1 — Supplementary info [file 41598_2019_51480_MOESM1_ESM.pdf]

Supplementary Information

Lignan enriched fraction (LRF) of *Phyllanthus amarus* promotes apoptotic cell death in human cervical cancer cells *in vitro*

Subhabrata Paul[1], Debashis Patra[2], Rita Kundu[3*]

Cell line maintenance

All the cell lines, HeLa, SiHa and C33A were procured from National centre for cell science (NCCS), Pune and maintained following American Type Culture Collection (ATCC) guidelines. HeLa, SiHa and C33A cell lines were maintained in Eagle's minimal essential medium (EMEM) with 10% Fetal bovine serum (FBS), 0.15% sodium bicarbonate, 2 mM L-glutamine, 100 units/ml penicillin, 100 µg/ml streptomycin and 250 ng/ml Amphotericin B at 37°C in a humidified incubator (Thermo Scientific) having 5% Carbon dioxide. NIH-3T3 cell line was maintained with Dulbecco's Modified Eagle Medium (DMEM) with 10% Fetal calf serum (FCS), other supplements and culture conditions remained same.

Evaluation of cytotoxicity

Cytoxicity of the LRF was checked by MTT assay (Mossman) and the results were reported in the previous publication[1]. IC50 doses in different cell lines were calculated and all the following experiments were performed with those doses (FigS1.)

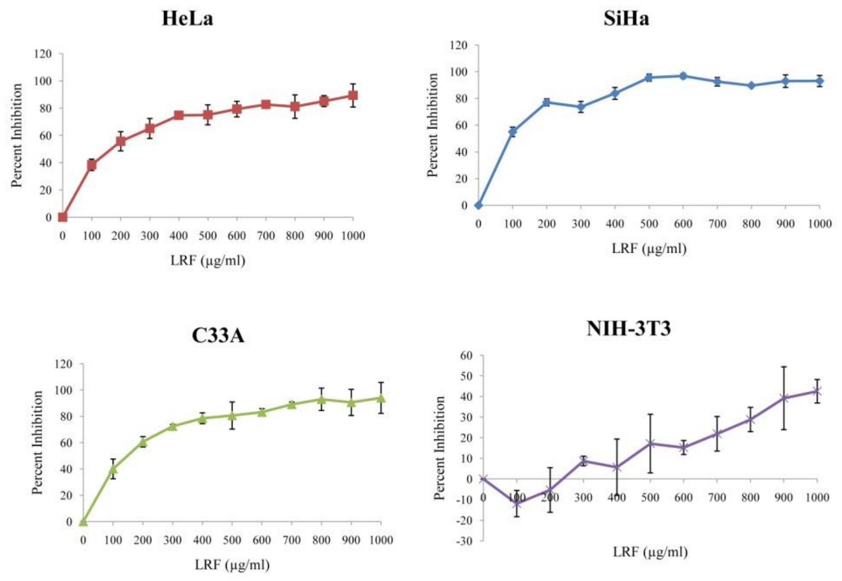

Fig. S1: MTT assay of LRF in different cell lines

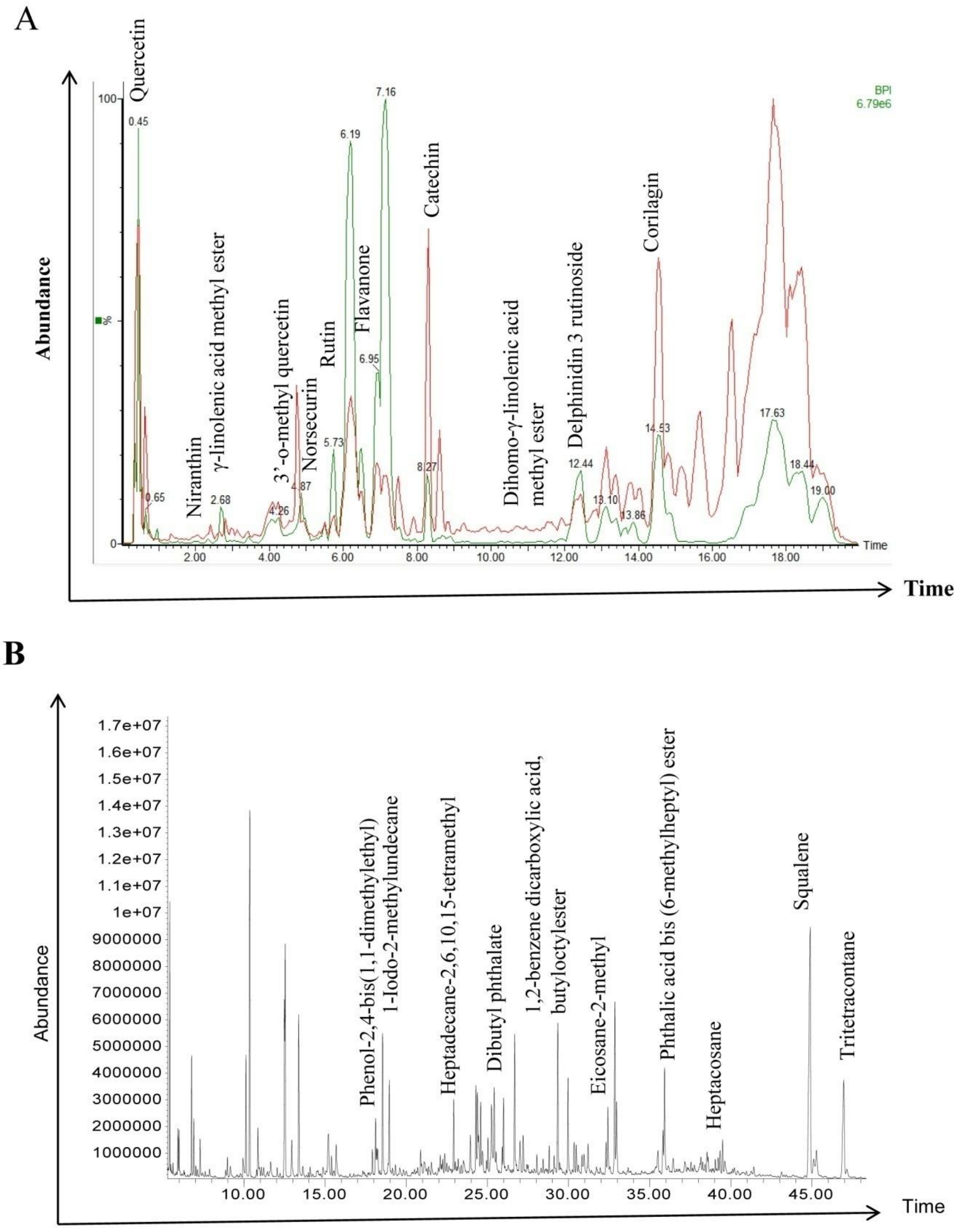

Fig.S2 Chromatogram from LC-MS (A) and GCMS (B) analysis. Green and red lines correspond to low and high energy chromatograms respectively.

Table S1. Phytochemicals present in the LRF found by LC-MS and GC-MS analysis.

**LC-MS**		**GC-MS**		
**RT**	**Probable compound**	**RT**	**Probable compound**	**Area %**
0.45	Quercetin	18.181	Phenol-2,4-bis(1,1-dimethylethyl)	1.491
2.02	Niranthin	18.750	1-Iodo-2-methylundecane	1.218
2.38	γ-linolenic acid methyl ester	23.504	Heptadecane-2,6,10,15-tetramethyl	1.707
4.41	3’-o-methyl quercetin	25.969	Dibutyl phthalate	1.584
5.50	Norsecurin	27.808	1,2-benzene dicarboxylic acid, butyloctylester	3.691
5.73	Rutin	31.705	Eicosane-2-methyl	1.31
6.95	Flavanone	37.773	Phthalic acid bis (6-methylheptyl) ester	6.285
8.27	Catechin	40.012	Heptacosane	2.519
10.51	Dihomo-γ-linolenic acid methyl ester	45.534	Squalene	50.34
12.44	Delphinidin 3 rutinoside	47.047	Tritetracontane	3.636
14.53	Corilagin			

Table S2 XRD analysis of Phyllanthin crystal

**Bond precision**	C-C = 0.0035 A	
**Wavelength**	0.71073	
**Cell:** a=22.6071(8)	b=5.3538(2)	c=11.0907(4)
**Temperature**	294 K	
	**Calculated**	**Reported**
Volume	1192.48(8)	1192.48(8)
Space group	C 2	C 2
Hall group	C 2y	C 2y
Moiety formula	C24 H34 O6	C24 H34 O6
Sum formula	C24 H34 O6	C24 H34 O6
Mr	418.51	418.51
Dx,g cm-3	1.166	1.166
Z	2	2
Mu (mm-1)	0.083	0.083
F000	452.0	452.0
F000’	452.24	
h,k,lmax	30,7,14	30,7,14
Nref	2953[1631]	2899
Tmin,Tmax	0.978,0.993	0.774,0.829
Tmin’	0.966	
Correction method= # Reported T Limits: Tmin=0.774 Tmax=0.829		
AbsCorr=	MULTI-SCAN	
Data completeness= 1.78/0.98	**Theta(max)= 28.243**	
R(reflections)= 0.0461(2540)	wR2(reflections)= 0.1421(2899)	
S = 1.060	Npar= 139	

Table S3 Different type of interactions between phyllanthin with different proteins obtained from molecular docking study.

Protein	VdW + H bond + dissolving energy	Electrostatic Energy	Total (1)	Final total internal energy (kcal mol^{-1}) (2)	Torsional free energy (kcal mol^{-1}) (3)	Unbound system's energy (4)	Estimated free energy of binding [(1) + (2) + (3) - (4)] (kcal mol^{-1})
**E6**	-8.22	-0.54	-8.76	-2.05	+3.88	-2.05	-4.89
**MDM**	-7.28	-0.06	-7.34	-1.01	+3.88	-1.01	-3.47
**p53**	+121.06	-0.57	+120.49	+6.03	+3.88	+6.03	+124.37

Table S4: WB and ICFC Specifications of antibodies, along with their respective dilutions.

**Protein**	**Manufacturer**	**Code**	**Reactivity**	**Dilution**	**Experiment**
Caspase-3	Cell Signaling Technology	9665	Rabbit	1:500	**Western Blot**
BAX	Santa Cruz Biotechnology	Sc-7480	Mouse	1:500	
Bcl-2		Sc-509			
p53		Sc-126			
p21		Sc-6246			
Caspase 8		Sc-52897			
Caspase 9	AbCam	Ab-32539			
HPV 16/18 E6		Ab-70			
ß-tubulin	Sigma	T8328	Mouse	1:5000	
p-ATR	Cell Signaling Technology	2853	Rabbit	1:500	
p-ATM		5883			
p-Chk1		2349			
p-Chk2		2661			
Goat anti-Rabbit IgG – AP	Santa Cruz Biotechnology	Sc-2007	Rabbit	1:5000	
Goat anti-Mouse IgG – AP	Enzo Life Science	ADI-SAB-101	Mouse		

γ-H2AX	Cell Signaling Technology	9718	Rabbit	1:50	**Indirect Immuno fluoresence**
Goat anti-Rabbit IgG- FITC	Sigma	F0382	Rabbit		
Caspase-3 PE	BD Biosciences	550821	Rabbit	20µl/10^6 cells	**Intracellular flow cytometry**
PARP PE		552933	Mouse		
BAX- Alexa fluor 488	Biolegend	633603	Mouse	1µg/10^6 cells	
Bcl-2 PE	Miltenyl Biotech	130-105-473	Mouse	1:10 for 10^6 cells	
p53 PE		130-109-570			

Table S5: Primer sequences and PCR profiles of the genes analyzed in RT-PCR

**Gene**	**Primer sequence (5’→ 3’)**	
	**Forward**	**Reverse**
**p53**	ATGGCCATCTACAAGCAG	ACAGTCAAGAGCCAACCTCAG
**p21**	ATGAAATTCACCCCCTTTCC	CCCTAGGCTGTGCTCACTTC
**BAX**	GTGGCAGCTGACATGTTTTC	GGAGGAAGTCCAATGTCCAG
**Bcl-2**	GGGTACGATAACCGGGAGAT	CTGAAGAGCTCCTCCACCAC
**ET-1**	TCCTCTGCTGGTTCCTGACT	CAGAAACTCCACCCCTGTGT
**HPV 16 E6**	TTGCTTTTCGGGATTTATGC	CAGGACACAGTGGCTTTTGA
**HPV 18 E6**	TGAAAAACGACGATTCCACA	TTGTGTTTCGCGTCGTT
**GAPDH**	CAAGGTCATCCATGACAACTTTG	GTCCACCACCCTGTTGCTGTAG

	**PCR profile**						
	**Initial denaturation (°C- minutes)**	**Denaturation (°C - seconds)**	**Annealing (°C - seconds)**	**Extension (°C - seconds)**	**Number of cycles**	**Final extension (°C- minutes)**	**Product length (bp)**
**p53**	94 – 4	94-30	58 - 30	72 - 45	35	72 – 7	210
**p21**			58 - 30	72 - 30			174
**BAX**			58 - 30	72 - 30			151
**Bcl-2**			58 - 30	72 - 45			395
**ET-1**			57 - 30	72 - 45			242
**HPV 16 E6**			52 - 30	72 - 30			106
**HPV 18 E6**			53 - 30	72 - 30			285
**GAPDH**			58 - 30	72 - 45			496

Fig S3. Fluorescent micrographs of annexin V-FITC/PI stained LRF treated HeLa, SiHa and C33A cells.

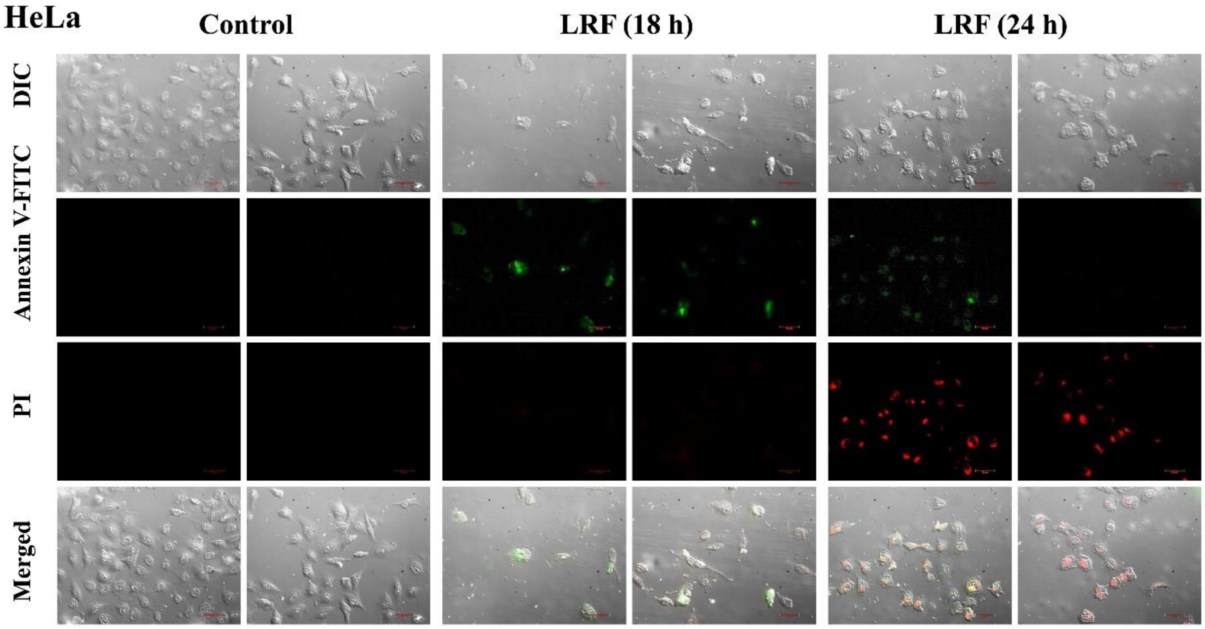

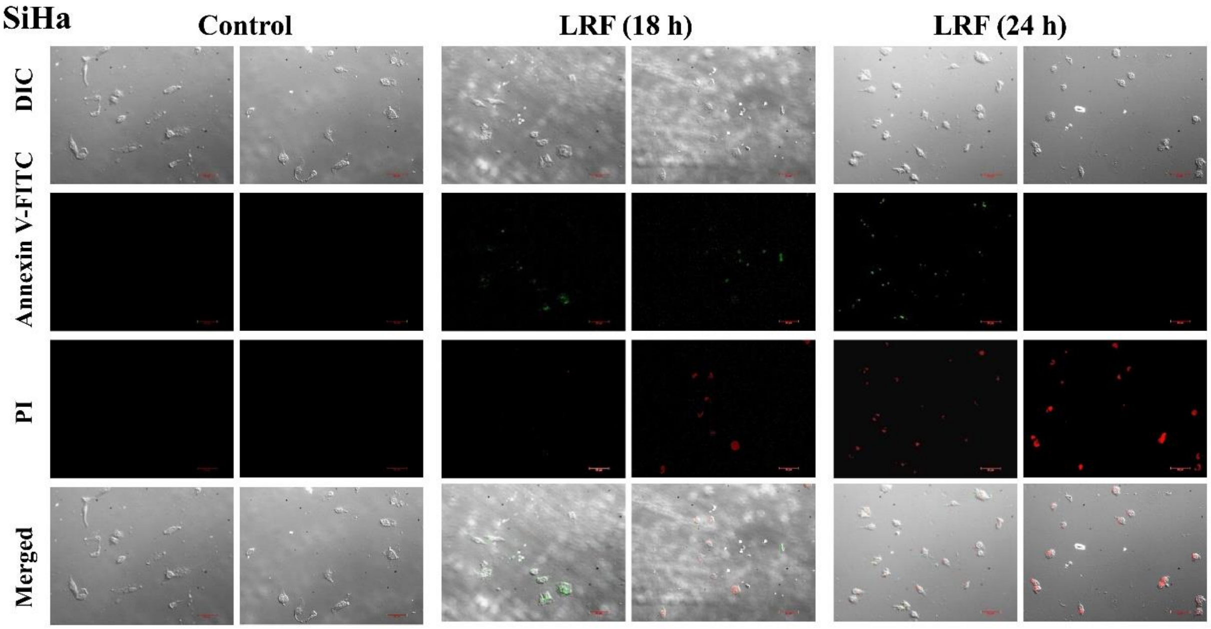

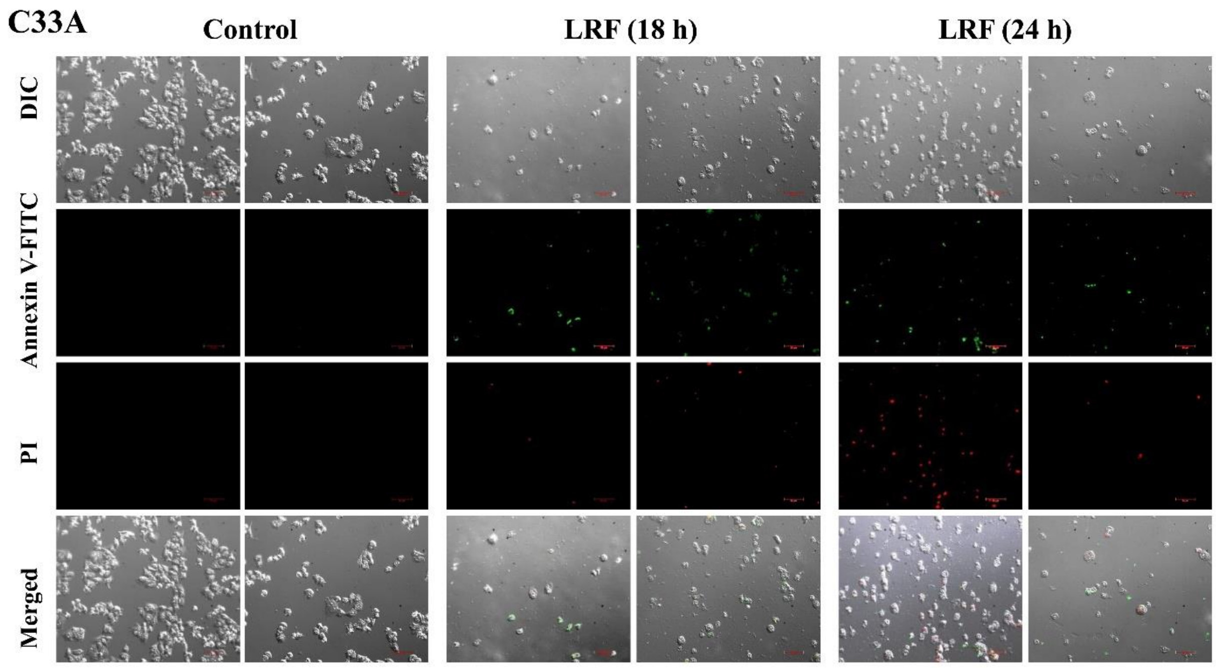

Fig. S4. No. of fragmented or condensed nuclei per 100 cells in the control and LRF treated sets

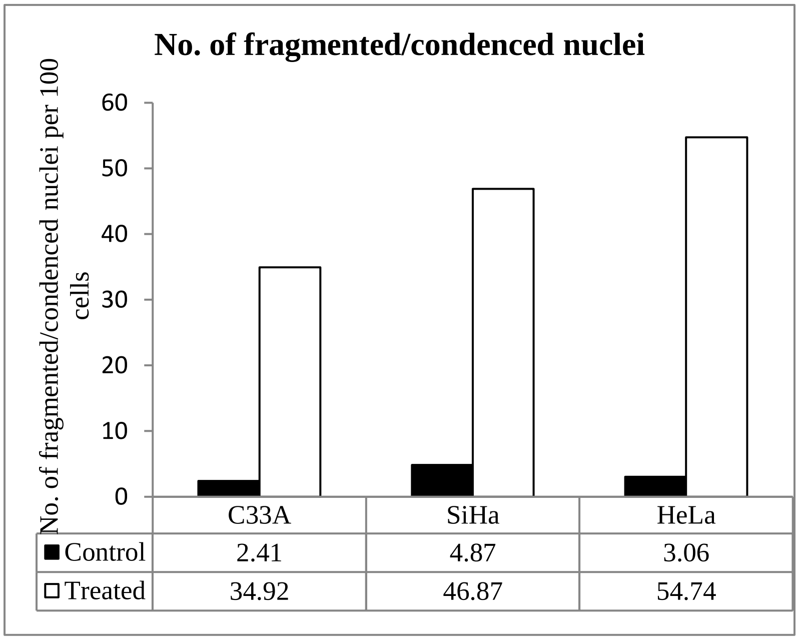

	C33A	SiHa	HeLa
■ Control	2.41	4.87	3.06
□ Treated	34.92	46.87	54.74

Additional microscopic pictures

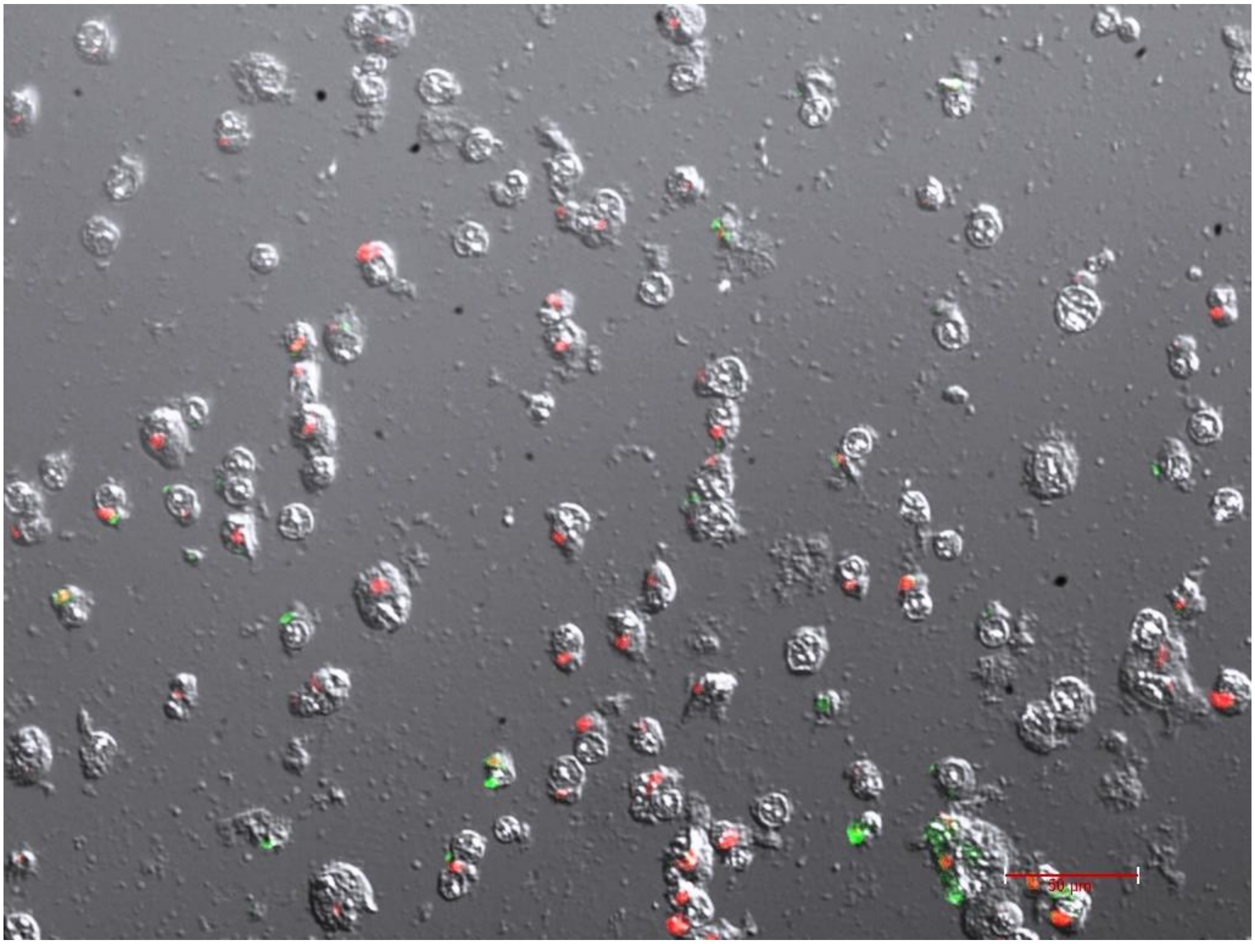

LRF treated C33A cells

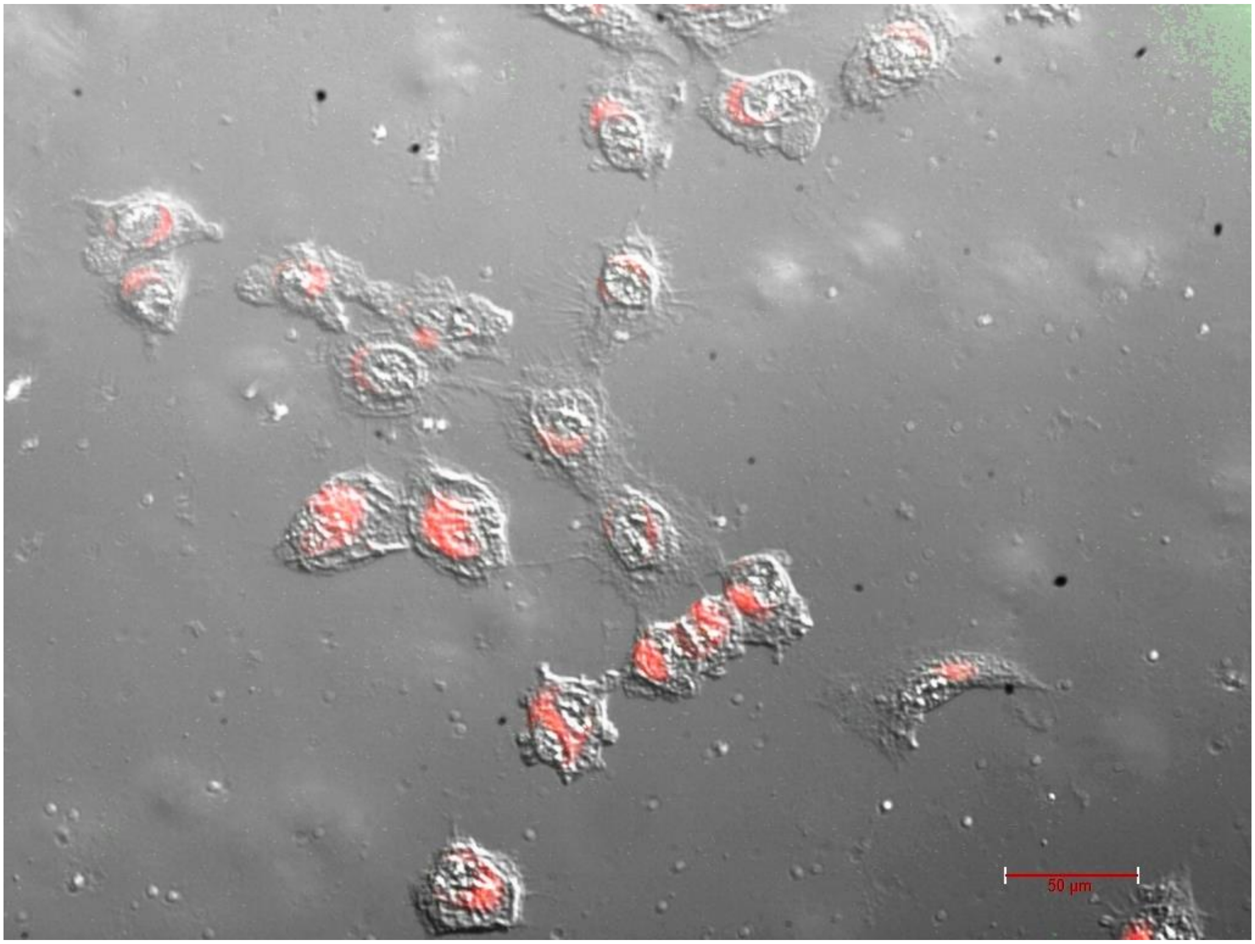

LRF treated HeLa cells

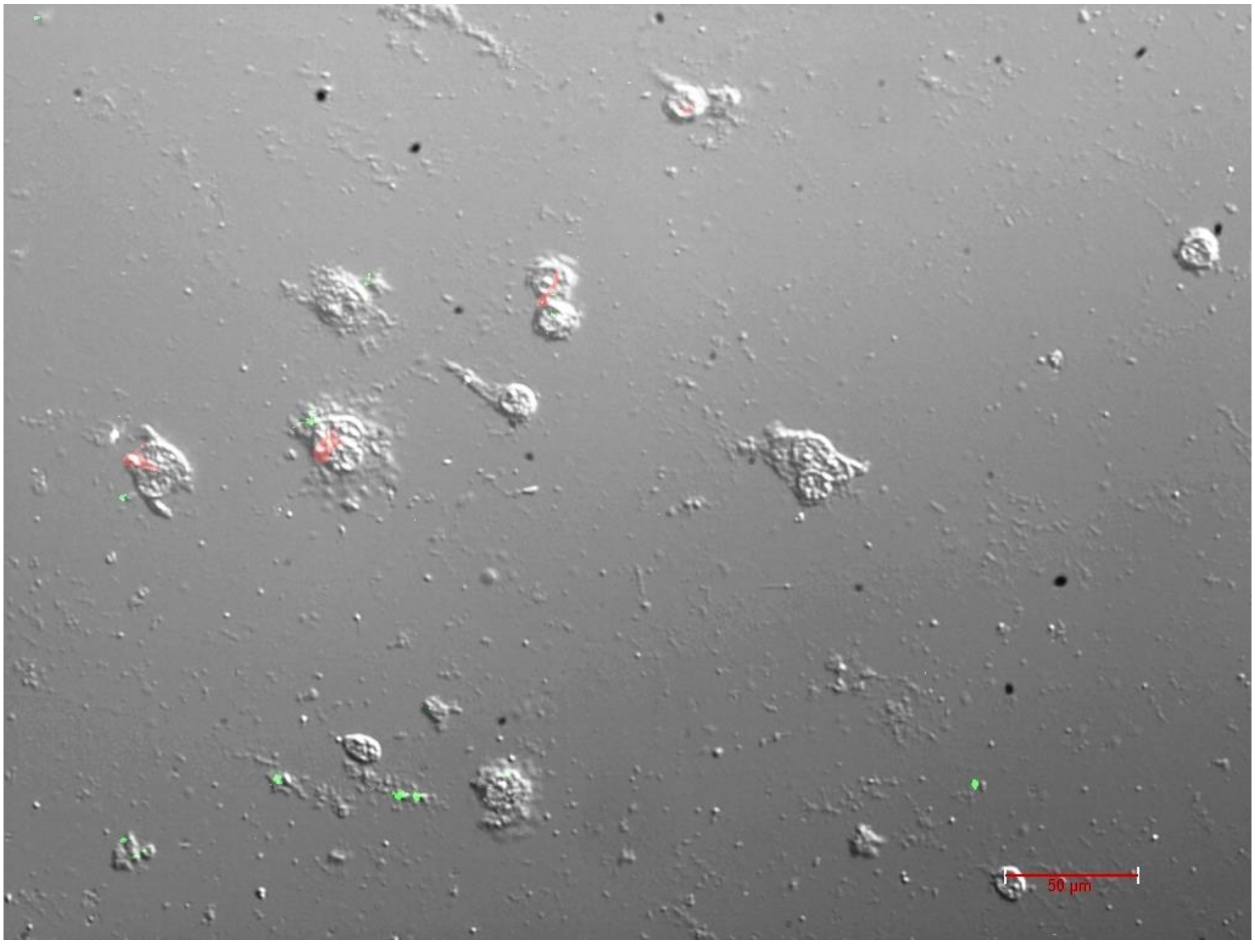

LRF treated SiHa cells

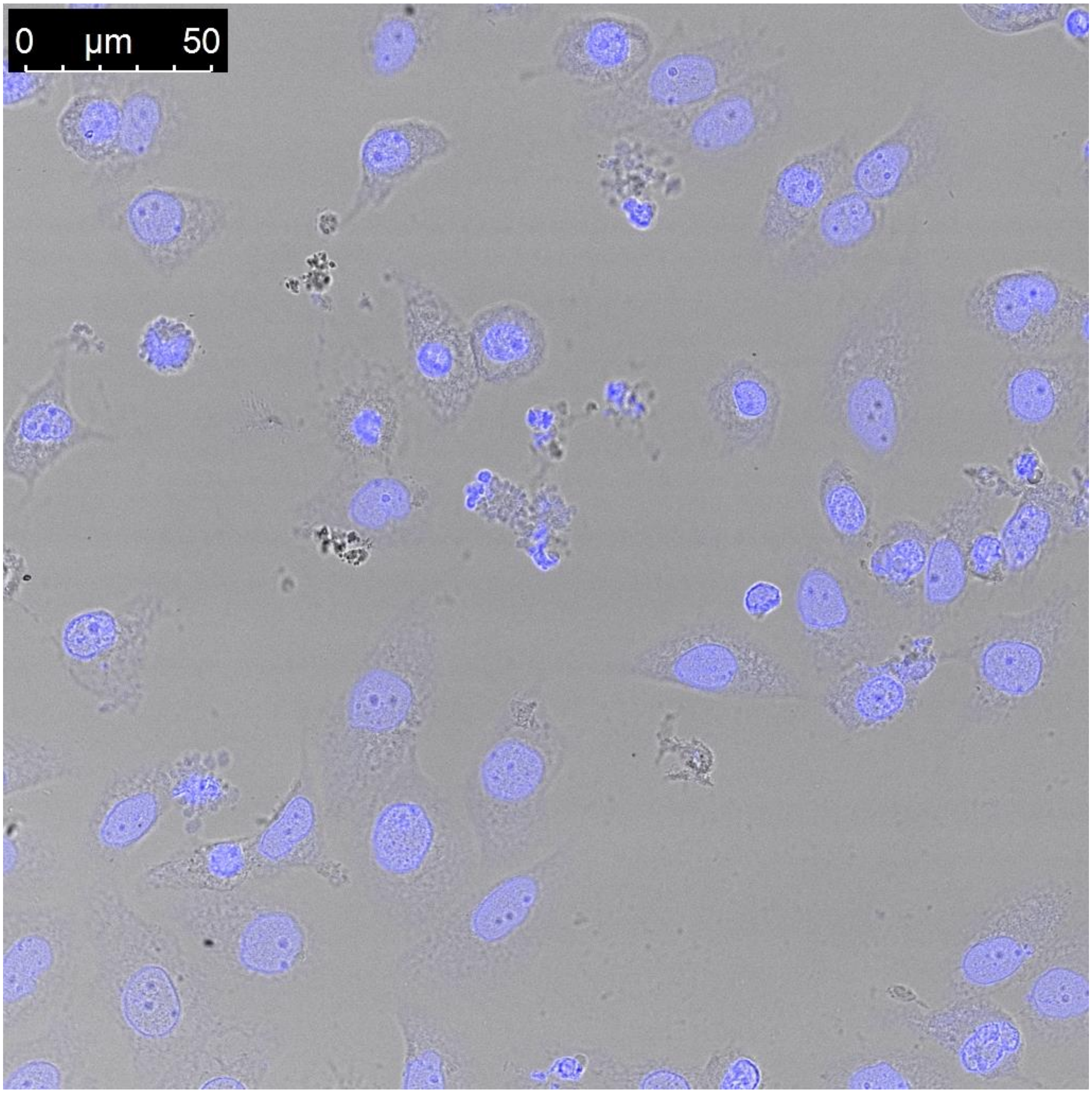

LRF treated SiHa cells

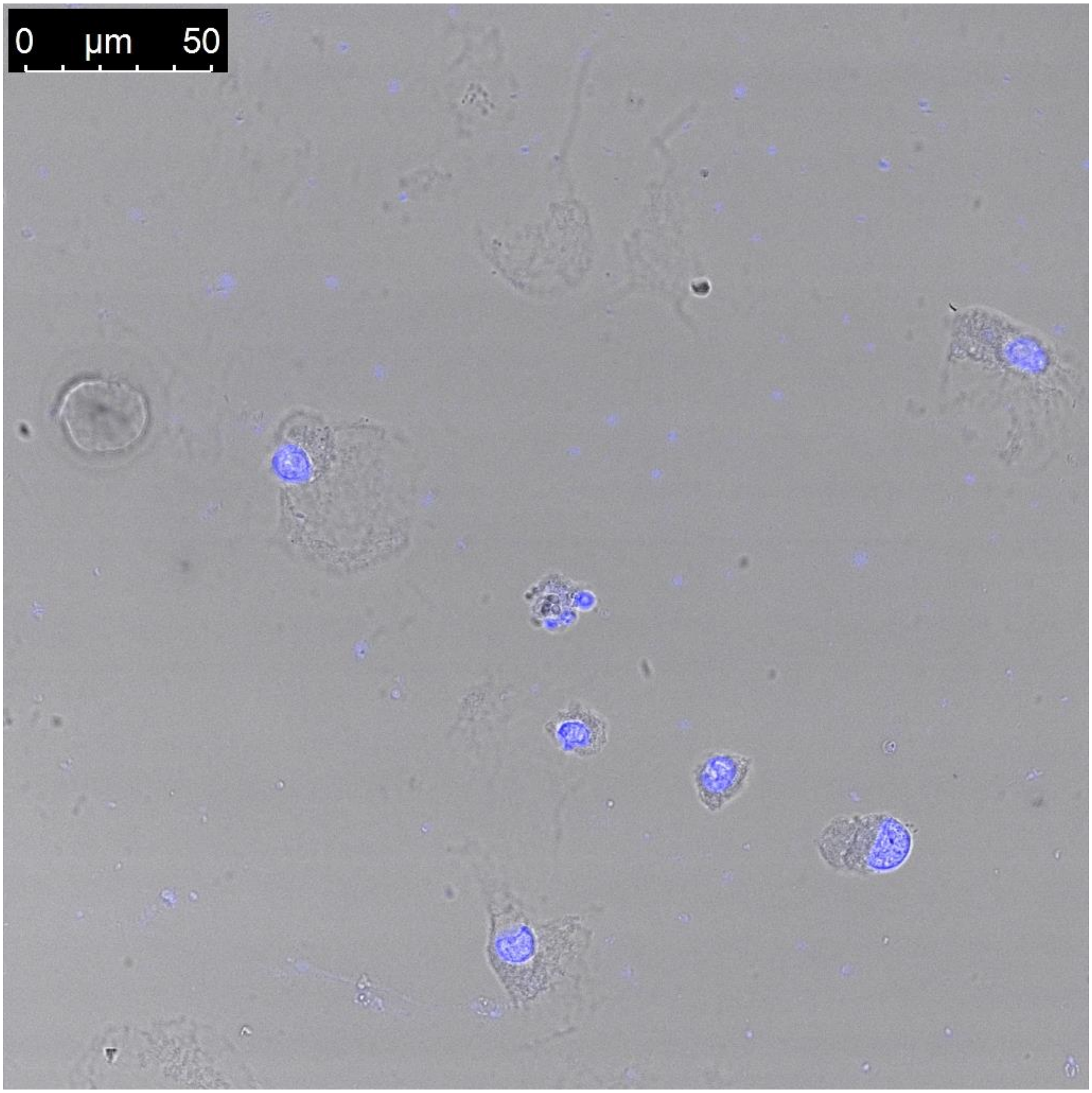

LRF treated HeLa cells

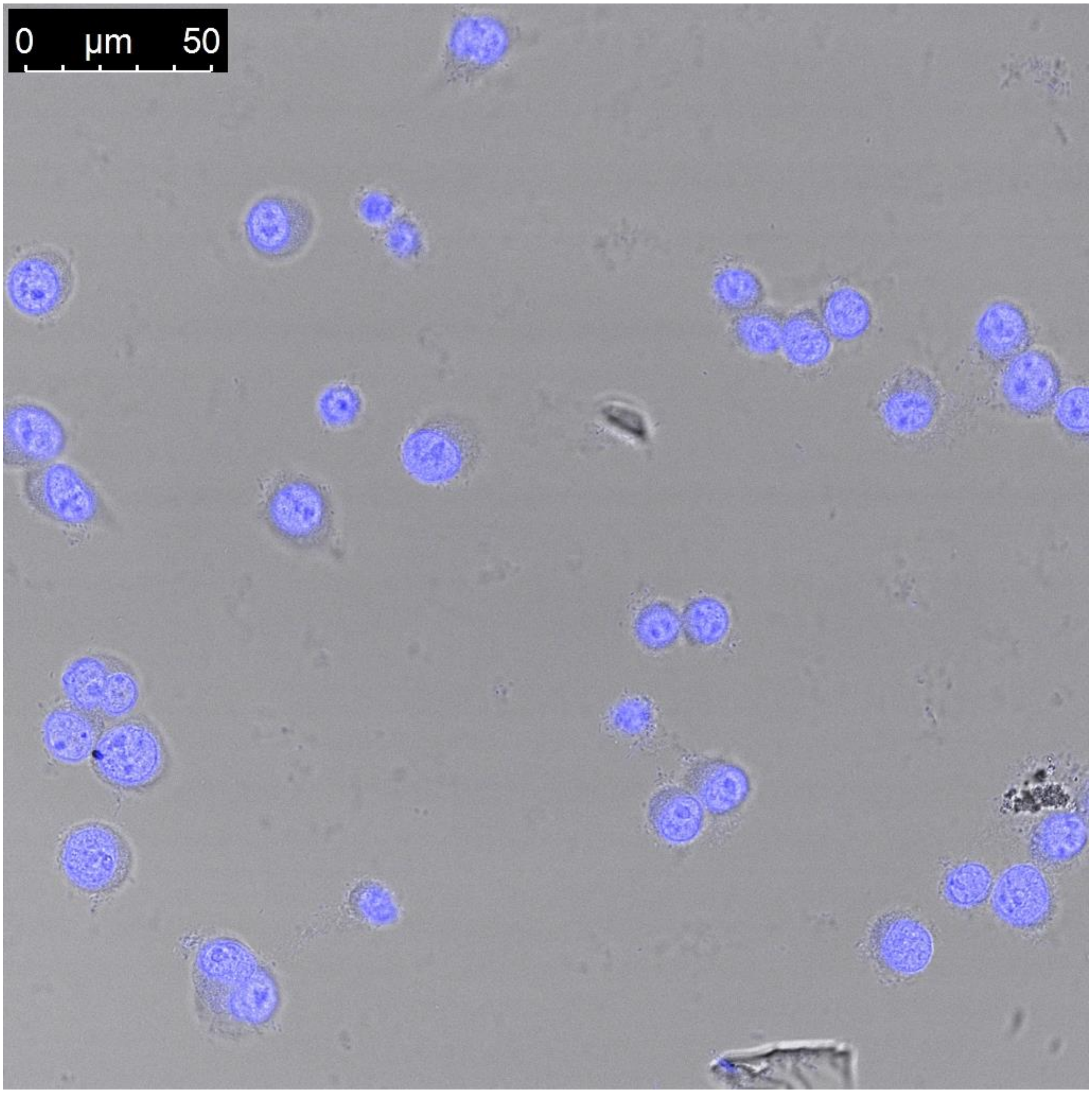

LRF treated C33A cells

Additional Gel pictures

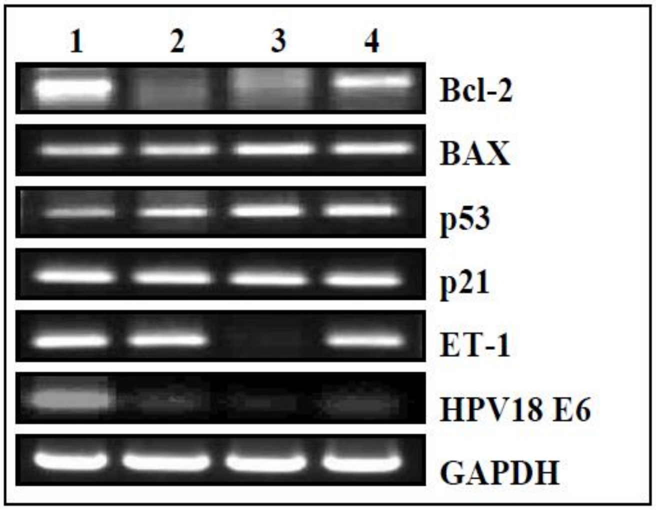

RT-PCR in HeLa cells. Lane 1: Control set, Lane 3: LRF treated sets.

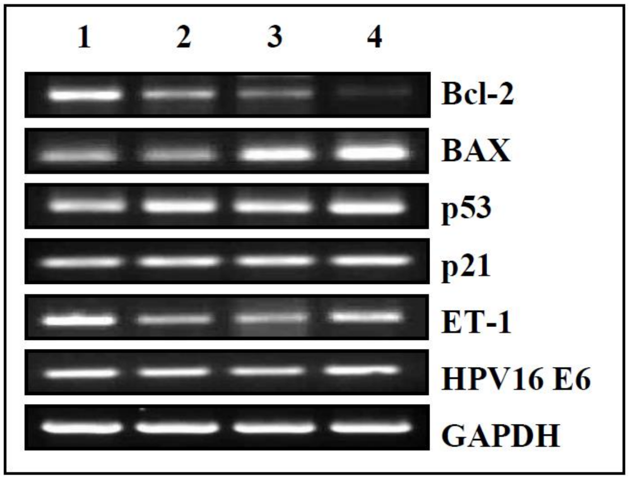

RT-PCR in SiHa cells. Lane 1: Control set, Lane 3: LRF treated sets.

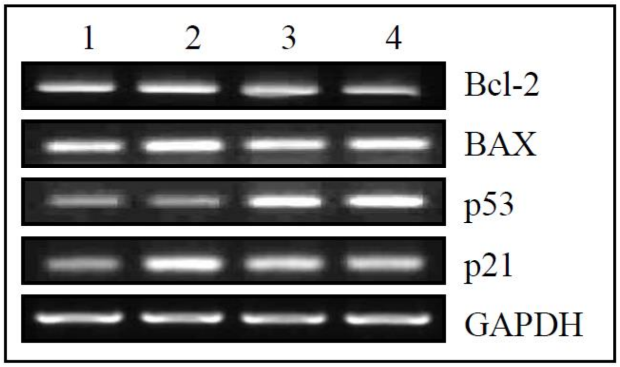

RT-PCR in C33A cells. Lane 1: Control set, Lane 3: LRF treated sets.
